# Culture Media for Woody Plant Tissue Culture: From Classical Formulations to Rational Optimization

**DOI:** 10.3390/plants15213366

**Published:** 2026-10-30

**Authors:** Sameena Ejaz Tanwir, Keila E. Rodriguez, Beth Lamb, Michael E. Kane, Heqiang Huo

**Affiliations:** 1 Crop Transformation Center, University of Florida, Gainesville, FL 32611, USA; tanwir.s@ufl.edu (S.E.T.); keilarodriguez@ufl.edu (K.E.R.); 2 Mid Florida Research and Education Center, Institute of Food and Agricultural Sciences, University of Florida, Apopka, FL 32703, USA; 3 Department of Horticultural Sciences, University of Florida, Gainesville, FL 32611, USA; micropro@ufl.edu; 4 Phillip Rucks Nursery, Frostproof, FL 33843, USA

**Keywords:** plant tissue culture, woody plants, basal media, media formulation, micropropagation, regeneration, somatic embryogenesis, plant growth regulators, recalcitrance, hyperhydricity

## Abstract

Plant tissue culture is essential for clonal propagation, germplasm conservation, genetic transformation, and genome editing in woody plants, yet these species remain highly challenging in vitro due to developmental recalcitrance, phenolic oxidation, endogenous contamination, seasonal effects, hyperhydricity, rooting difficulty, and strong genotype dependence. Medium formulation is therefore often a decisive factor rather than a routine technical step. This review examines the development, composition, and application of tissue culture media for woody species, emphasizing how specialized formulations emerged to overcome limitations of general-purpose media such as Murashige and Skoog medium. Major woody-targeted media, including Woody Plant Medium, Quoirin and Lepoivre medium, Anderson’s medium, Driver and Kuniyuki Walnut medium, Rugini Olive Medium, Litvay medium, and DCR medium, are discussed in relation to the physiological needs of their target species. Key medium components are reviewed, including mineral nutrients, carbon sources, vitamins, plant growth regulators, organic additives, gelling agents, and pH, with attention to nitrogen form, ionic strength, calcium supply, iron availability, and pathway-dependent plant growth regulator selection. The review further highlights applications in conservation, micropropagation, transformation, and genome editing, and discusses emerging approaches such as statistical optimization, machine learning, nanoparticles, temporary immersion systems, biostimulants, and morphogenic regulators.

## 1. Introduction

Plant tissue culture has become an indispensable platform for plant propagation, conservation, genetic improvement, and biotechnology. By providing aseptic, defined, and controllable conditions for the culture of cells, tissues, organs, or embryos, in vitro systems enable rapid clonal multiplication, pathogen elimination, germplasm preservation, and regeneration after genetic transformation or genome editing [1,2,3,4]. At the center of every tissue culture system is the culture medium, which supplies mineral nutrients, carbon, vitamins, plant growth regulators, organic supplements, water, and physical support. Although often treated as a standard recipe, the medium is better understood as a developmental environment: its ionic strength, nitrogen form, calcium availability, carbon source, hormone balance, gelling properties, and pH collectively influence whether an explant remains viable, forms callus, regenerates shoots, develops somatic embryos, roots, or fails [2,5,6,7].

The importance of medium composition is especially evident in woody plant species. Trees, shrubs, woody climbers, and woody ornamentals include many of the world’s most economically and ecologically important plants, including fruit and nut crops, forest trees, ornamental shrubs, plantation species, and rare or endangered taxa. Efficient in vitro systems are therefore critical for their clonal propagation, germplasm conservation, breeding support, transformation, and genome editing [8,9,10]. However, woody plants remain among the most recalcitrant materials in plant tissue culture. Protocols that work efficiently for herbaceous models often perform poorly when transferred to woody taxa, particularly when mature elite genotypes are used as explant sources [11,12,13].

This difficulty reflects the distinctive biology of woody plants. Phenolic oxidation, endogenous contamination, mature-tissue (phase-related) recalcitrance, seasonal variability, hyperhydricity, shoot-tip necrosis, poor rooting, and strong genotype dependence interact in woody explants and are treated in detail in Section 3 [11,12,13,14,15,16,17,18,19,20]. These interacting barriers explain why media formulation for woody species has remained highly empirical and why universal protocols are rare [8,12].

Historically, plant tissue culture media were developed through progressive empirical refinement. Early mineral solutions such as Knop’s medium provided the nutritional foundation for in vitro culture, but sustained tissue growth required more complex and concentrated formulations [21,22]. The development of Murashige and Skoog medium represented a major breakthrough because it supported rapid growth of tobacco callus and became broadly useful across many plant species [5]. However, MS medium was optimized for tobacco callus growth [5] and, at full strength, supplies about 60 mmol/L total nitrogen (39.4 mmol/L NO_3_^−^ and 20.6 mmol/L NH_4_^+^, an NO_3_^−^:NH_4_^+^ ratio of about 1.9) with a macronutrient ionic strength of about 56 mmol/L (Supplementary Table S1) [5,22]. These characteristics are advantageous in some fast-growing cultures but can be excessive for many woody tissues [22,23]. The limitations of MS medium stimulated the development of alternative formulations better suited to woody species, including Quoirin and Lepoivre medium for *Prunus*, Woody Plant Medium for mountain laurel and other woody taxa, Anderson’s medium for *Rhododendron* and ericaceous species, Driver and Kuniyuki Walnut medium for *Juglans*, Rugini Olive Medium for *Olea europaea*, and conifer-oriented media such as Litvay and DCR [24,25,26,27,28,29,30].

The development of these woody-targeted media illustrates an important principle: basal media are not interchangeable salt mixtures, but physiological solutions to particular culture problems [7,22,23]. Reduced ammonium and lower ionic strength are often beneficial for ericaceous shrubs, conifers, and other salt-sensitive woody species [23,25,26]. Elevated calcium can reduce shoot-tip necrosis in woody species such as quince [31], and a high calcium supply is a defining feature of DKW medium for walnut [27]. Modified iron chelation has been reported to alleviate chlorosis in iron-sensitive cultures [32]. Carbon source, vitamin composition, cytokinin type, auxin type, gelling agent, and pH can also shift developmental outcomes [2,22,33]. Thus, successful media design requires more than selecting MS, WPM, DKW, or another basal formulation; it requires understanding how individual components interact with explant type, genotype, developmental phase, culture stage, and regeneration pathway [7,8,22]. In particular, plant growth regulator selection must be matched to the developmental pathway employed-callus induction, direct organogenesis, indirect organogenesis, somatic embryogenesis, or rooting-because auxin type, cytokinin type, and concentration jointly bias morphogenesis toward different developmental outcomes [2,6,33].

This review examines advances and trends in media formulations for woody plant tissue culture from historical, physiological, and practical perspectives. Section 2 traces the historical development of plant tissue culture media from early mineral solutions to specialized woody formulations. Section 3 discusses the biological barriers that make woody species distinct from herbaceous models, including phenolic oxidation, endogenous contamination, mature-tissue recalcitrance, seasonal variation, hyperhydricity, and genotype dependence. Section 4 reviews the major basal media used for woody species and the rationale behind their formulation. Section 5 analyzes the roles of individual medium components, including macronutrients, micronutrients, carbon sources, vitamins, plant growth regulators, organic additives, gelling agents, and pH; Section 5.9 integrates these components into a practical framework for selecting and modifying a basal medium for a new woody species or a recalcitrant genotype. Section 6 considers how optimized media support conservation, commercial micropropagation, genetic transformation, and genome editing. Section 7 highlights emerging approaches, including statistical and machine-learning-assisted optimization, nanoparticles, temporary immersion systems, natural biostimulants, and morphogenic-regulator-assisted regeneration. Together, these sections provide a framework for rational media selection and optimization in woody species, while also identifying knowledge gaps that must be addressed to improve reproducibility, scalability, and regeneration efficiency.

## 2. Historical Development of Plant Tissue Culture Media

**Figure 1:**
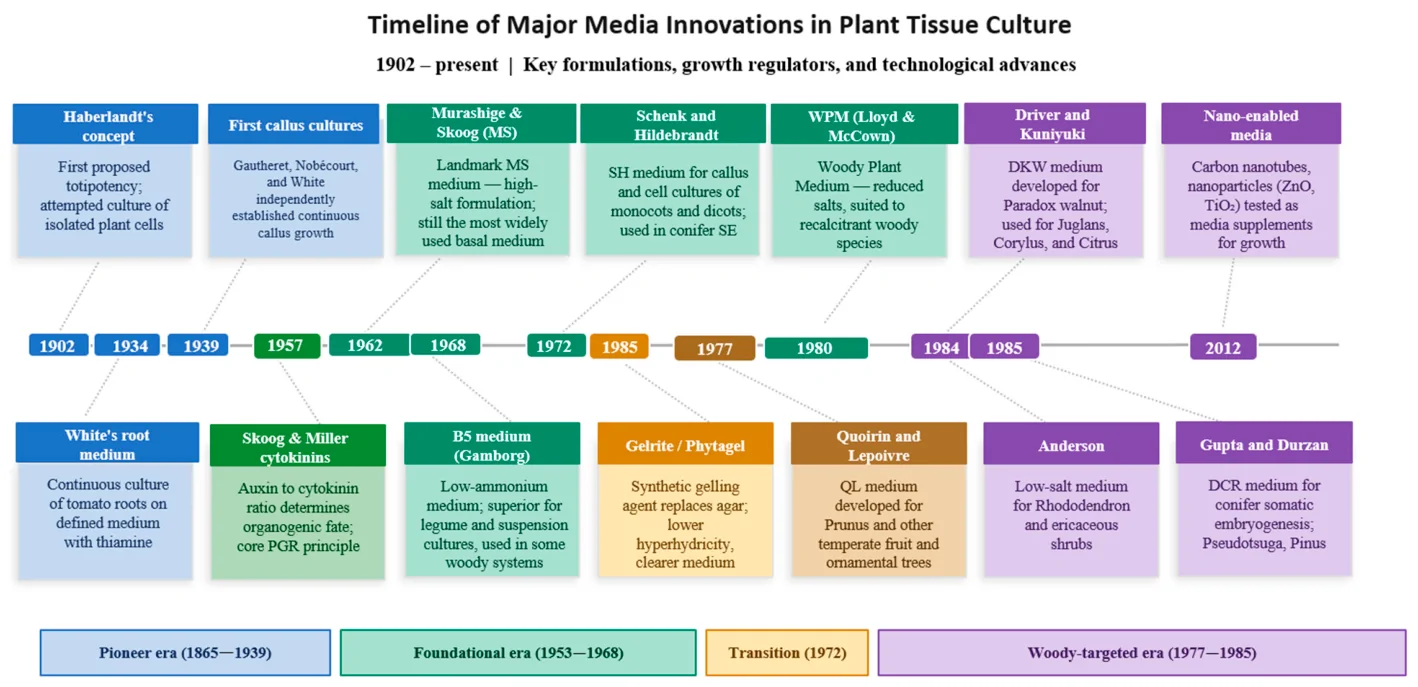
Timeline of major media innovations in plant tissue culture (1902-present), showing key formulations, growth regulators, and technological advances across Pioneer, Foundational, Transition, and Woody-targeted eras.

The evolution of plant tissue culture media reflects more than a century of empirical refinement in response to a recurring problem: a medium that supports one species, tissue type, or developmental pathway often fails in another. Each major formulation arose to address specific limitations of earlier media, and the formulations that later became important for woody species, including WPM, DKW, Anderson’s medium, and QL medium, were developed for particular taxa, tissue types, or physiological constraints. This section traces that trajectory from early mineral nutrient solutions through the MS breakthrough to the woody-targeted formulations that frame the rest of this review (Figure 1).

### 2.1. Early Developments in Plant Tissue Culture Media

The conceptual foundation of plant tissue culture preceded the development of any successful culture medium. In 1902, the Austrian botanist Gottlieb Haberlandt presented his classic experiments on the culture of isolated plant cells to the Vienna Academy of Sciences and articulated the principle of cellular totipotency—the idea that a plant cell, under appropriate conditions, could regenerate into a whole plant [34,35]. Although Haberlandt’s own cultured cells failed to divide, his vision profoundly shaped the direction of the field.

The mineral framework on which later culture studies were built had been established earlier. Knop’s solution, originally formulated for hydroponic culture of whole plants, provided one of the earliest defined mineral bases for plant growth and later served as a foundation for in vitro experimentation [21,22]. Haberlandt himself used a Knop-based solution supplemented with sucrose. In the early twentieth century, Hannig successfully cultured isolated crucifer embryos on dilute mineral salts with sugar, while Robbins and Kotte independently established some of the first organ cultures by growing pea and maize root tips on simple defined media [36,37,38].

A major advancement came with the work of Philip R. White, who reported continuous culture of excised tomato (*Lycopersicon esculentum*) roots on a medium containing inorganic salts, sucrose, and the B vitamin thiamine [39]. In 1939, Gautheret and Nobécourt in France and White in the United States independently established continuously growing callus cultures of carrot and tobacco, demonstrating the indefinite proliferation of dedifferentiated plant tissues in vitro [40,41,42,43]. However, these early successes still relied on complex organic supplements such as yeast extract, casein hydrolysates, or coconut water, whose active components had not yet been fully identified [1,44].

Subsequent studies showed that sustained tissue growth required higher ionic strength and more balanced mineral nutrition than whole-plant hydroponic solutions. Heller demonstrated that increasing the salt concentration of Knop’s solution improved growth of carrot and Virginia creeper tissues [45]. Nitsch and Nitsch further showed that explants of Jerusalem artichoke (*Helianthus tuberosus*) required higher inorganic salt concentrations for optimal tissue proliferation, and that the balance between nitrate and ammonium was as important as total salt concentration [46]. These studies established that in vitro tissues are not simply miniature whole plants, but systems with distinct nutritional requirements. This recognition directly motivated the development of more concentrated and more precisely balanced media in subsequent decades.

A parallel and equally influential line of research clarified the role of plant growth regulators. In their seminal experiments with tobacco pith, Skoog and Miller demonstrated that the auxin-to-cytokinin ratio, rather than the absolute concentration of either hormone alone, strongly influences whether an explant forms roots, shoots, or undifferentiated callus [6]. High auxin-to-cytokinin ratios promoted rooting, low ratios favored shoot organogenesis, and intermediate ratios maintained callus growth. This principle remains a cornerstone of organogenic media design and, as discussed in later sections, often requires substantial recalibration when applied to woody species.

### 2.2. The Breakthrough: Murashige and Skoog (MS) Medium

The MS medium, published by Murashige and Skoog (1962), was developed by systematically optimizing inorganic salt composition for tobacco callus growth based on tissue ash analysis [5]. This formulation marked a major breakthrough in plant tissue culture because it provided a well-defined medium that supported rapid and reproducible growth across many species. Compared with earlier formulations such as Knop’s solution, MS medium contains a substantially higher total salt concentration, with nitrogen supplied as both nitrate (NO_3_^−^) and ammonium (NH_4_^+^), together with an expanded set of micronutrients, including iodine and molybdenum [5,22]. Combined with sucrose, vitamins, myo-inositol, and appropriate plant growth regulators, these features made MS medium highly effective for the culture of many herbaceous plants, and it remains the most widely used basal medium in plant tissue culture today [22].

In woody species, its high total ionic strength, high nitrogen concentration, and relatively high ammonium content can be excessive or poorly tolerated, particularly in slow-growing perennial tissues [21,23,25,27,47,48]. These limitations motivated the development of alternative basal media better suited to woody plant culture, as described in the following subsection.

### 2.3. Specialized Media Formulations for Woody Plants

By the late 1960s and 1970s, it had become evident that no single culture medium could meet the requirements of all plant species, and a series of new formulations emerged to address specific limitations (Figure 1). Gamborg’s B5 medium, originally formulated for soybean (*Glycine max*) cell suspension cultures rather than woody plants, introduced lower ammonium levels and a modified vitamin composition that were later adopted in many plant tissue culture protocols, including some woody plant systems sensitive to ammonium toxicity [49]. Similarly, Schenk and Hildebrandt developed SH medium for callus and suspension cultures of both monocots and dicots; although not intended specifically for woody taxa, it was subsequently applied in some conifer somatic embryogenesis systems [50].

The first formulations developed specifically for woody species appeared in the late 1970s and early 1980s. Quoirin and Lepoivre introduced a modified MS-derived medium, now commonly known as QL medium, for *Prunus* meristem and shoot cultures; the formulation reduced ionic strength and adjusted nitrogen composition to improve culture performance in woody rosaceous species [24]. QL medium has since been adopted for *Prunus*, *Pyrus*, *Malus*, *Rosa*, and other temperate fruit and ornamental woody species. Anderson developed a low-salt medium with reduced ammonium and a modified iron composition for *Rhododendron* and other ericaceous shrubs that often perform poorly on MS-based media [26]. Anderson’s medium has also been widely used for *Vaccinium* and *Rubus* species [51,52].

Lloyd and McCown introduced Woody Plant Medium (WPM), originally developed for mountain laurel (*Kalmia latifolia*) [25]. In WPM, potassium nitrate was replaced by potassium sulfate, ammonium nitrate was reduced relative to MS medium, and a large proportion of nitrogen was supplied as calcium nitrate. WPM has since become one of the most widely used basal media for woody species, including members of *Malus*, *Prunus*, *Populus*, *Rhododendron*, and other woody genera [25]. Litvay medium, derived from analytical data on developing conifer seeds, was formulated for conifer cell suspension and somatic embryogenesis cultures and remains relevant for embryogenic systems in *Picea*, *Pinus*, and *Pseudotsuga* [29].

Driver and Kuniyuki developed DKW medium for Paradox walnut rootstock (*Juglans hindsii* × *J. regia*) [27]. DKW medium provides higher calcium and magnesium than MS or WPM and has proven effective for *Juglans*, *Corylus*, and several other woody species [27,53]. Rugini introduced Olive Medium (OM), enriched in zinc, magnesium, calcium, and sulfur and supplemented with glutamine, which became a standard formulation for *Olea europaea* and other recalcitrant Mediterranean woody species [28]. Conifer somatic embryogenesis prompted further specialization, exemplified by DCR medium, developed by Gupta and Durzan for conifer tissue culture and now widely used in *Picea* and *Pinus* embryogenic culture systems [30].

Three observations emerge from this history and frame the rest of this review. First, major woody-targeted formulations were developed to address specific limitations of general-purpose media in particular woody taxa or developmental systems. WPM reflects the requirements of *Kalmia* and related woody species; DKW reflects the demands of walnut; Anderson’s medium reflects the requirements of ericaceous shrubs; and QL medium reflects the needs of *Prunus* and related woody rosaceous species. The choice of basal medium for a new woody species should therefore be informed by phylogenetic and physiological proximity to the species or tissue system for which each medium was originally optimized. Second, despite decades of use, relatively few woody plant media have been fundamentally redesigned. Modern protocols more often modify ionic strength, adjust plant growth regulator composition, or add organic supplements than redesign the basal salt composition. Third, the historical trajectory has been overwhelmingly empirical: formulations were optimized for specific tissues by trial and error, whereas the underlying physiological reasons that some taxa benefit from reduced ammonium, lower ionic strength, or elevated calcium remain incompletely understood. Recent advances in plant ionomics, metabolomics, and data-driven optimization offer the prospect of a more mechanistic basis for media design, a theme developed in later sections of this review.

## 3. Why Woody Species Are Different: Biological Barriers to In Vitro Culture

The specialized media described in Section 2 were developed largely as empirical responses to the poor performance of woody tissues on general-purpose formulations such as MS medium. Behind these failures are distinctive physiological, anatomical, and developmental features that separate woody plants from the herbaceous models on which many classical tissue culture systems were established. Trees and shrubs are typically more highly differentiated, more lignified, richer in phenolic compounds, more seasonally variable, and more strongly affected by ontogenetic age than herbaceous species [54,55]. These features influence not only explant survival but also callus formation, shoot regeneration, somatic embryogenesis, rooting, and acclimatization [56,57]. This section summarizes the major biological barriers that shape woody-species tissue culture and provides the rationale for the basal media and component-level modifications discussed in Section 4 and Section 5.

### 3.1. Recalcitrance and the Interactivity of Barriers

**Figure 2:**
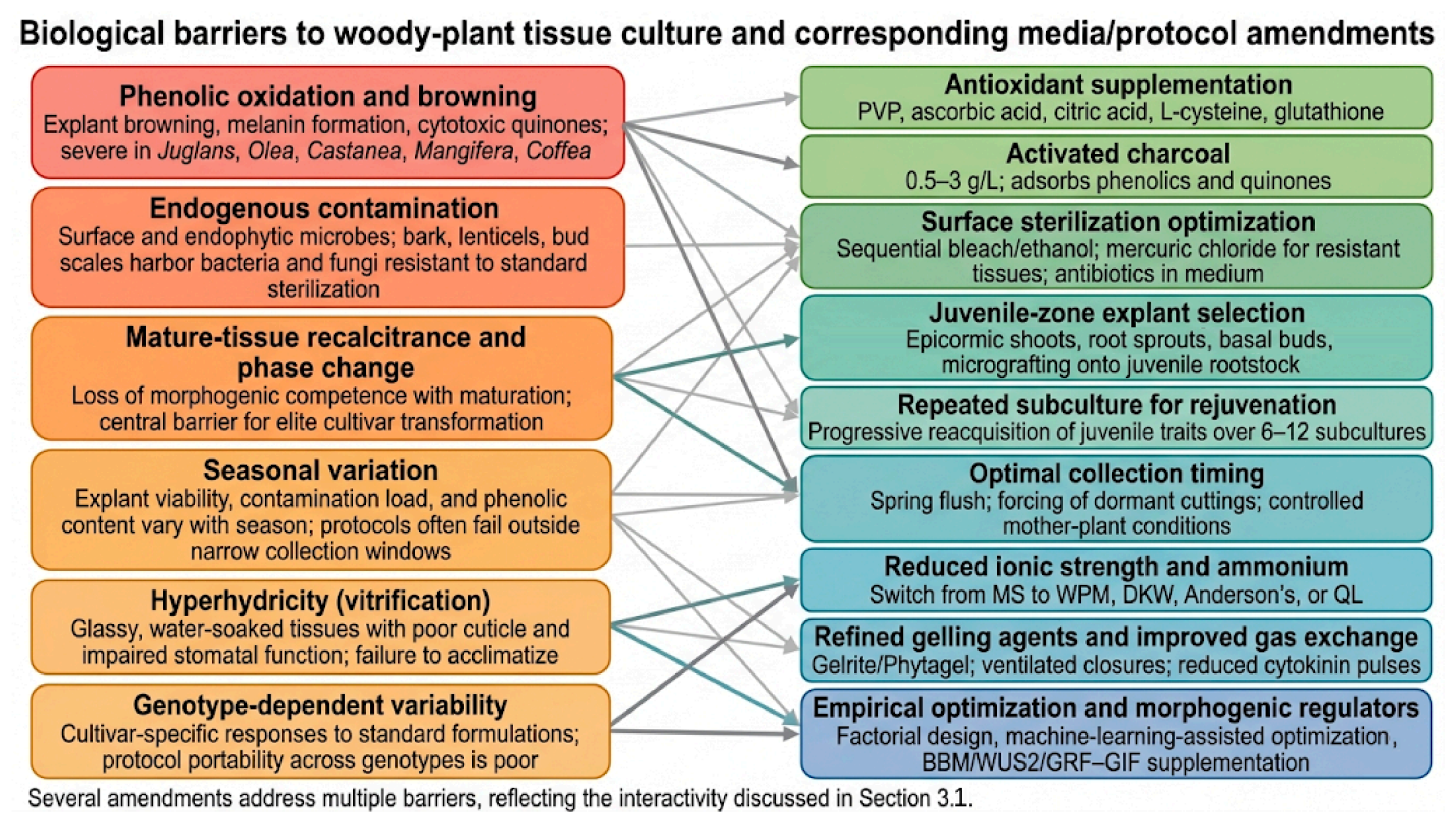
Biological barriers to woody-plant tissue culture (**left**) and the corresponding media and protocol amendments developed to address them (**right**). Each barrier connects to one or more amendments via arrows. Several amendments—including optimal collection timing, surface sterilization optimization, reduced ionic strength, and empirical optimization with morphogenic regulators—address multiple barriers simultaneously, reflecting the interactivity of recalcitrance factors discussed in Section 3. Frequent subculture and dark culture, additional interventions for phenolic browning, are described in Section 3.2 but omitted here for visual clarity.

Woody-species recalcitrance is best understood as the combined effect of several interacting barriers (Figure 2) rather than as a single limitation [54,55]. Common constraints include phenolic oxidation [14], endogenous contamination [57,58], mature-tissue recalcitrance [11,59,60], seasonal variation [61], hyperhydricity [20], and genotype-dependent variability [62]. These barriers frequently reinforce one another. For example, aggressive sterilization used to control contamination can damage tissues and increase phenolic leakage [14,63]; mature explants often have both lower regeneration competence and higher phenolic content [59,60]; and the high cytokinin concentrations used to stimulate shoot formation in recalcitrant genotypes can also promote hyperhydricity [20]. This interdependence explains why woody-species protocols are difficult to standardize and why media optimization often remains empirical [54,57]. A successful protocol usually requires coordinated adjustment of explant source, collection timing, sterilization intensity, basal medium, plant growth regulators, gelling system, and culture environment [11,55,64,65].

### 3.2. Phenolic Oxidation and Tissue Browning

Phenolic oxidation is among the most persistent obstacles in the establishment of woody plant cultures. It typically appears as browning of the explant and surrounding medium and, in severe cases, leads to explant death within days of inoculation. The biochemical basis is well established: woody tissues, particularly bark, cambium, and woody stems, accumulate high concentrations of phenolic compounds, including hydroxycinnamic acids, flavonoids, condensed tannins, and lignin precursors, as part of normal secondary metabolism and constitutive defense [14,66]. Wounding during explant excision releases these compounds and exposes them to polyphenol oxidases and peroxidases, which catalyze their oxidation to quinones. These quinones can polymerize to form melanin-like pigments responsible for the characteristic browning and can react with proteins and nucleic acids to produce cytotoxic adducts that compromise tissue viability [14].

Several lines of evidence indicate that browning severity is not a fixed species property but is modulated by the physiological state of the explant, the surface sterilization regime, and the medium composition [67,68]. Because phenolics are products of secondary metabolism, browning severity is also related to the physiological age of the explant: mature tissues generally contain more phenolics and brown more readily than juvenile material [11,59,60], and the transition to the mature phase is under developmental regulation, including the miR156-SPL pathway [13] (see Section 3.4). Excessive sterilization, particularly with strong oxidants such as hypochlorite, can exacerbate phenolic accumulation in the medium, likely by damaging cell membranes and promoting phenolic leakage [14,63]. Phenolic exudation also varies seasonally, with several woody species, including *Ceratonia siliqua* and *Corylus avellana*, showing greater browning during autumn or winter and reduced browning during spring or active growth periods [15,58,69].

**Figure 3:**
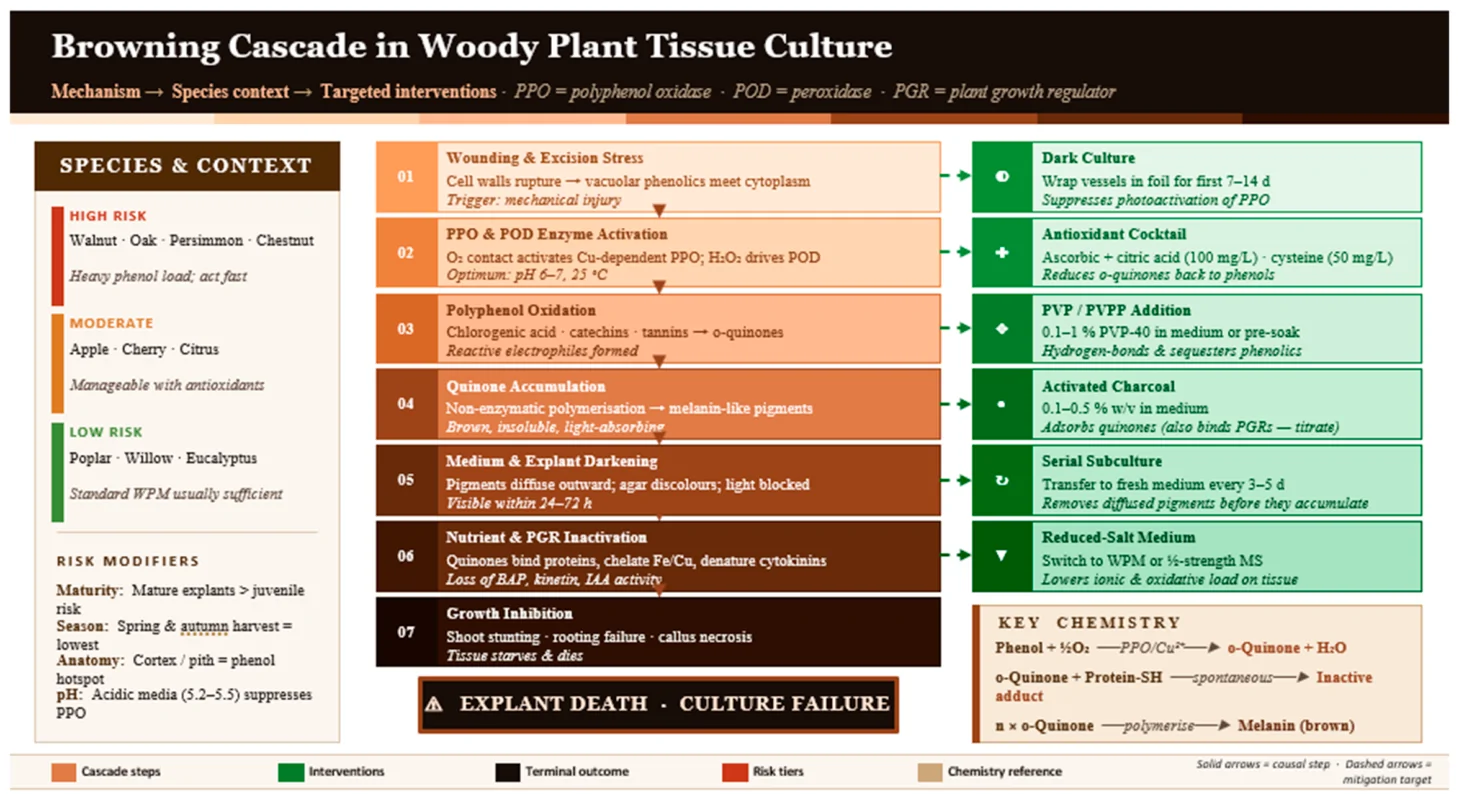
Browning cascade in woody plant tissue culture. Wounding releases phenolic compounds that are oxidized by polyphenol oxidases (PPOs) and peroxidases (PODs) to reactive *o*-quinones. These quinones can polymerize into melanin-like pigments, interfere with nutrient and plant growth regulator (PGR) availability, damage cellular components, and contribute to explant decline or culture failure. Relative browning risk is shown from high to low: walnut/oak/persimmon/chestnut → apple/cherry/citrus → poplar/willow/eucalyptus, and is modulated by explant maturity, season, tissue anatomy, genotype, and medium pH. Intervention strategies target different steps in the cascade, including dark culture, antioxidant supplementation, PVP/polyvinylpolypyrrolidone (PVPP), activated charcoal, frequent transfer to fresh medium, and reduced-salt formulations.

The toolkit for managing phenolic browning in woody tissue culture has been built incrementally over several decades and now comprises several strategies that are typically used in combination rather than alone. Antioxidant supplementation, including ascorbic acid, citric acid, L-cysteine, glutathione, or polyvinylpyrrolidone (PVP), can reduce oxidation of phenolics in the medium and on the explant surface [14]. Adsorbents, principally activated charcoal, can sequester phenolics and quinones from the medium, although activated charcoal can also adsorb plant growth regulators and therefore requires corresponding adjustments in hormone concentration [64]. Frequent subculture during the establishment phase removes accumulated oxidation products and provides fresh medium before phenolic concentrations become inhibitory. Dark or low-light culture during the first several days of establishment can also reduce light-stimulated phenolic metabolism and is commonly used in woody-plant protocols. Recent work has explored more sophisticated strategies, including controlled antioxidant delivery and the use of silver nitrate to modulate ethylene-associated oxidative responses [14]. The biochemical cascade linking wound-induced phenolic oxidation to explant decline, together with species-specific risk and intervention points, is summarized in Figure 3.

Despite the long list of available interventions, browning remains a major bottleneck in establishing new woody-species protocols. The reason is not that individual interventions are ineffective, but that their performance is highly species-, explant-, and context-dependent. There is no general rule that predicts whether *Olea*, *Juglans*, *Citrus*, or another phenolic-prone genus will respond best to antioxidant pretreatment, PVP, activated charcoal, dark culture, frequent subculture, or a combination of these approaches. Protocol development therefore requires empirical testing of treatment combinations, and the mechanistic basis for species-specific differences remains incompletely understood.

### 3.3. Surface Disinfection and Control of Endogenous Contamination

Woody plants present substantially greater contamination challenges than herbaceous species, and the reasons are anatomical and ecological rather than merely procedural. The bark, cambium, buds, lenticels, and vascular tissues of trees and shrubs can harbor diverse communities of endophytic bacteria and fungi that occupy internal tissue spaces and are not removed by surface sterilization [57,63,70]. Long-lived perennial tissues accumulate microbial residents over years or decades, and field-grown explants are exposed to weather, dust, and insect activity that can deposit microbes into bark fissures, lenticels, and bud scales. Reported contamination rates in woody-plant tissue culture vary widely depending on species, season, and source material, and latent endophytes may emerge only after several weeks of subculture [57,58,70,71].

Two distinct problems should be separated. Surface contamination is caused by epiphytic microorganisms on bark, bud scales, and lenticels and is addressed by surface disinfection of the explant (ethanol followed by hypochlorite or sodium dichloroisocyanurate (NaDCC) with a surfactant) before culture initiation. Endogenous contamination is caused by endophytic bacteria and fungi inside the tissue, which surface disinfection cannot reach; it is managed through the choice of clean donor material and collection season, pretreatment of stock plants, antimicrobial supplements in the medium, and early indexing of established cultures [57,63,70]. Heat sterilization, including autoclaving, is used for media, instruments, and vessels but is not a method for disinfecting explants, because it kills plant tissue.

The standard surface sterilization regime-ethanol pretreatment followed by sodium hypochlorite with surfactant-is generally effective for soft, juvenile herbaceous tissues but often inadequate for woody explants. Several modifications have therefore been adopted for woody species. Mercuric chloride has historically been used in some laboratories because of its strong disinfectant activity, but its toxicity and disposal concerns make it undesirable for routine use. Sequential sterilization, involving repeated exposures to bleach or alcohol with intervening rinses, can improve contamination control but often increases tissue damage and phenolic leakage. NaDCC is a chlorine-releasing disinfectant widely used for surface sterilization of seeds and explants; like sodium hypochlorite, it acts through free available chlorine, but it is often more stable in solution. The following measures address endogenous rather than surface contamination. Antibiotic and fungicide supplementation of the medium, including compounds such as cefotaxime and timentin, can suppress endophytes that survive surface sterilization, although chronic antimicrobial use may suppress rather than eliminate contaminants and may also affect explant growth [71,72]. Thermotherapy or pretreatment of mother plants has shown effectiveness in reducing endogenous microbial loads in some woody species, including hazelnut, by limiting systemic infection before culture initiation [58].

More recently, contamination management strategies have shifted toward antimicrobial agents incorporated directly into the culture medium to provide sustained microbial suppression during the establishment phase. Plant Preservative Mixture, a broad-spectrum biocide, has been widely adopted as an alternative or complement to conventional antibiotics [72,73]. Nevertheless, accumulating evidence indicates that such preservatives do not always eliminate endophytic microorganisms, and tolerant populations may persist or re-emerge during subsequent subcultures [70,72]. Low-concentration oxidants have also been explored as continuous antimicrobial agents in culture media. Sodium hypochlorite at very low concentrations can suppress microbial growth during early culture stages while reducing the severe phytotoxic effects associated with conventional sterilization treatments [63]. Hydrogen peroxide offers a complementary mechanism through oxidative damage to microbial membranes and nucleic acids [74].

Beyond oxidant-based approaches, metal nanoparticles have attracted growing interest as medium additives. Silver nanoparticles and zinc oxide nanoparticles have shown antimicrobial and growth-modulatory effects in several in vitro systems, although concentration-dependent phytotoxicity remains a major limitation [75,76]. Chlorine dioxide has also been explored as a medium sterilant or surface treatment, with reported effects on both microbial suppression and adventitious shoot organogenesis in at least one ornamental system [77]. Essential oils represent another emerging category; rosemary essential oil, for example, has been reported to suppress microbial contamination and oxidative browning simultaneously in shoot cultures [78]. Collectively, these approaches reflect a broader conceptual shift from attempting to achieve complete sterility toward maintaining controlled microbial suppression, a strategy especially relevant for woody species where endophytic colonization is extensive and persistent.

Two practical observations recur across the woody-species literature. First, sterilization regimes optimized for one season, cultivar, or source plant may fail when applied to material collected at a different time or from a different genotype because endophyte loads vary with environment and physiological state. Second, the trade-off between sterilization severity and tissue viability is particularly acute for woody species because aggressive sterilization can release more phenolics, compounding the browning problem described in Section 3.2. Many woody-plant protocols therefore combine moderate surface sterilization with antimicrobial supplementation rather than attempting to achieve sterility through chemical treatment alone. Emerging nanoparticle- and oxidant-based strategies are best viewed as complements to this multi-component framework rather than replacements for it.

Forcing solutions used to precondition woody explants typically contain plant growth regulators (especially cytokinins like 6-benzylaminopurine (BAP)), antioxidants, and sometimes fungicides, applied to donor plants or cut shoots to stimulate bud break and enhance meristematic activity prior to explant establishment [79]. This pretreatment improves the physiological status of the tissue by reducing dormancy and increasing cellular competence, leading to better explant survival and reduced oxidative browning during initiation [80,81]. Consequently, preconditioned explants exhibit significantly improved regeneration responses and contamination-free establishment in micropropagation systems for recalcitrant woody species [82,83].

### 3.4. Mature-Tissue Recalcitrance, Juvenility, and Phase Change

Among the barriers discussed in this section, mature-tissue recalcitrance is one of the most consequential for transformation and genome editing and one of the least amenable to correction by medium formulation alone. Woody plants pass through a juvenile phase of variable duration, during which vegetative growth is vigorous and tissues often retain relatively high capacity for adventitious organogenesis and rooting. With the transition to the mature reproductive phase, the same tissues frequently lose much of this morphogenic competence, a process variously termed maturation, phase change, ontogenetic aging, or cyclophysis [54,56,59].

This transition is not strictly chronological but ontogenetic. Tissues at the base of a mature tree may retain juvenile characteristics, such as epicormic shoots or basal sprouts, whereas tissues in the canopy often express mature characteristics more strongly [11,59]. The biological basis of phase change involves epigenetic regulation, changes in chromatin state, shifts in endogenous plant growth regulator balance, and conserved regulatory pathways such as the miR156-SPL module, in which the microRNA miR156 declines with age and thereby releases SQUAMOSA PROMOTER BINDING PROTEIN-LIKE (SPL) transcription factors that promote the transition to the adult phase [84]. Despite progress in understanding these mechanisms, the molecular basis of mature-tissue recalcitrance remains only partially resolved [60]. In citrus, for example, constitutive expression of the *Arabidopsis* genes *LEAFY* or *APETALA1* shortened the juvenile phase and reduced generation time [85].

Juvenile and mature explants differ in several properties that bear directly on medium design. Juvenile material, such as seedling tissues, basal sprouts, and epicormic shoots, typically establishes with less browning and contamination, responds to lower plant growth regulator doses, and roots readily. Mature, crown-derived material more often requires stronger cytokinin regimes (for example, thidiazuron (TDZ) pulses), proliferates more slowly and variably, and roots poorly even after prolonged subculture [11,54,55,59]. The age-related decline in morphogenic competence is associated with changes in endogenous hormone balance, epigenetic state, and the miR156–SPL module [84], none of which is corrected by changing basal salts. For elite mature genotypes, this has three consequences. First, responses of seedlings or rejuvenated shoots cannot be assumed to predict the behavior of mature material. Second, experiments should include a juvenile reference genotype and state the ontogenetic origin of each explant, so that effects of physiological age are not mistaken for genotype or medium effects. Third, rejuvenation or the use of juvenile zones of mature plants should be combined with medium optimization rather than replaced by it [11,55,86].

As a result, protocols developed for seedlings or in vitro-derived juvenile material often fail when applied to elite mature genotypes-the very genotypes that breeders, transformation programs, and gene-editing pipelines most need to access. In citrus, conifers, walnut, oak, apple, and many other woody species, regeneration efficiency from mature-derived explants is usually far lower than that from juvenile material, and in some systems mature tissues show little or no regeneration under tested conditions [55]. The implications for genome editing are particularly acute: delivery of editing reagents into mature tissue has limited value if edited cells cannot be recovered as whole plants.

Strategies for managing mature-tissue recalcitrance fall into several broad classes. Tissue selection from juvenile zones of mature plants, including basal sprouts, epicormic shoots, root suckers, and stump sprouts, provides juvenile-like material from genetically mature individuals and is one of the most reliable routes when available [11,55]. Rejuvenation through repeated subculture exploits the observation that shoots can gradually reacquire juvenile traits, including improved rooting and morphogenic competence, although this process is slow and highly genotype-dependent [11,54]. Micrografting onto juvenile rootstocks combines a mature scion with a juvenile rootstock and has been particularly useful in citrus and other woody crops [87,88,89]. More aggressive approaches, including somatic embryogenesis from mature tissues, induction of meristematic tissues with cytokinin pulses, and the use of morphogenic regulators, remain species-specific and variable in success [90].

A broader perspective is that efforts to overcome mature-tissue recalcitrance through media optimization alone have yielded useful but incremental improvements. Media composition strongly influences culture response, but under the conditions tested to date, no formulation has been shown to consistently restore mature tissues to juvenile-equivalent regenerative capacity across woody species. Developmental regulators such as BABY BOOM, WUSCHEL, GRF-GIF chimeras, and PLT5 represent complementary strategies that supply developmental signals genetically rather than chemically [65,91,92]. Whether these approaches can resolve woody-species recalcitrance at scale remains uncertain, but they suggest that future regeneration systems may need to combine rational medium design with engineered developmental competence.

Growth habit should also be considered when optimizing media for woody plants. In species with strong orthotropic (vertical) versus plagiotropic (lateral) shoot growth, the orientation and position of the explant on the donor plant, together with cytokinin type and concentration, can strongly affect shoot proliferation and the growth form of regenerated shoots in vitro [93].

### 3.5. Seasonal Variation and Explant Timing

Seasonal effects on explant performance are well documented in woody species but remain under-reported in the protocol literature. Because many published methods specify source plant and explant type but not collection season or physiological state, seasonal effects can become an invisible contributor to protocol irreproducibility. The underlying biology reflects coordinated seasonal shifts in carbohydrate reserves, endogenous plant growth regulator concentrations, secondary metabolite levels, microbial endophyte loads, tissue water status, and dormancy status [8,58,61].

Empirical studies in several woody species have documented strong seasonal effects. In *Pinus sylvestris*, callus proliferation from bud explants was highest during certain winter and active-growth periods and lowest during other seasonal windows; contamination rates also varied seasonally [61]. In *Ceratonia siliqua*, shoot establishment was optimal in spring, whereas phenolic exudation and contamination were higher during late autumn and winter [69]. In *Corylus avellana*, summer-collected explants yielded a higher proportion of clean, viable cultures, while autumn and winter material showed higher contamination rates associated with seasonal precipitation patterns and elevated bark microbial loads [58,62]. In *Eucalyptus* and many tropical woody species, active flush periods generally provide the most reliable window for obtaining soft, minimally lignified tissues suitable for explant initiation.

Three practical strategies have emerged for managing seasonal effects. Restricting explant collection to optimal seasons remains the simplest and most widely used approach; many woody-plant protocols rely on material collected during periods of active growth, typically late spring or early summer in temperate species [2,8]. Forcing dormant stem segments under controlled conditions can provide soft, cleaner shoots from material collected outside the natural flush window and has been useful for species whose natural flush is brief or unpredictable [2,8,94]. Maintenance of mother plants under controlled greenhouse or growth-room conditions, where photoperiod, temperature, irrigation, and nutrition are regulated, can also reduce or decouple seasonal variability and is widely used in commercial micropropagation.

From a methodological perspective, the routine omission of collection timing and physiological state from published protocols represents a small but consequential reporting gap. Protocols that perform reliably in one laboratory under specific seasonal conditions may fail elsewhere when those conditions differ. Greater transparency in reporting explant collection parameters, including season, phenological stage, and, where relevant, dormancy status, would improve reproducibility and facilitate cross-study comparison.

### 3.6. Hyperhydricity and Abnormal Morphogenesis

Hyperhydricity, historically referred to as vitrification, is a physiological disorder of in-vitro-grown shoots characterized by translucent, water-soaked tissues, reduced lignification, malformed leaves, hypertrophic stems, and impaired stomatal function. These abnormalities frequently result in poor or failed acclimatization because shoots have excessive water content, weak cuticular development, and limited stomatal functionality [19,20]. Although hyperhydricity occurs in herbaceous tissue cultures, it is particularly problematic in woody plant micropropagation, where it can substantially reduce the usability of regenerated shoots.

Hyperhydricity is a multifactorial physiological disorder rather than the result of any single cause, and it is best understood as the outcome of interactions among four groups of factors [19,20,95,96]. (i) Water availability: soft media or low gelling-agent concentrations make water readily available and favor excessive tissue hydration. (ii) Gas exchange: tightly closed vessels produce high relative humidity, suppress transpiration, and allow ethylene, CO_2_, and other volatiles to accumulate, which is associated with oxidative stress and impaired stomatal function. (iii) Mineral nutrition: high ammonium and high total salt concentrations alter osmotic and nitrogen metabolism, and calcium supply has also been implicated. (iv) Plant growth regulators: high concentrations of cytokinin, or of highly active cytokinins such as TDZ and BAP, promote the disorder, and altered auxin–cytokinin balance and hormone-related stress signaling, including ethylene, have been linked to it [97]. These factors interact: a cytokinin-rich medium that is tolerated on a firm gel in a ventilated vessel may produce hyperhydric shoots on a soft gel in a sealed vessel, and ammonium effects depend on pH and calcium supply. Physiologically, hyperhydricity is regarded as a stress-induced change in state in which oxidative stress, disturbed water relations, and altered cell-wall structure are common features [20,96]. Auxin imbalance is therefore one contributing component rather than the primary cause, and much of the evidence for individual factors comes from a limited number of species.

In woody species such as *Prunus*, *Malus*, *Pyrus*, and *Vaccinium*, hyperhydricity is frequently observed under high-cytokinin shoot multiplication conditions, particularly when cultures are maintained in poorly ventilated vessels [19,20]. At the cellular level, excessive water uptake relative to structural development is associated with reduced lignification and impaired differentiation of vascular and epidermal tissues, contributing to the characteristic glassy phenotype [20,96,98,99].

A range of mitigation strategies has been developed, typically applied in combination rather than isolation. These include reducing total ionic strength of the medium or replacing full-strength MS medium with lower-salt formulations such as WPM or Anderson’s medium, lowering ammonium concentration, reducing cytokinin concentration or exposure duration, increasing gel strength through higher agar concentration or alternative gelling agents, and improving vessel gas exchange through ventilated closures or controlled-aeration culture systems [19,20,25]. The benefit of lower-salt media is not universal, however: in black walnut, WPM and half-strength DKW produced hyperhydric shoots at frequencies of 60–100%, compared with about 10–40% on MS or full-strength DKW [100]. Antioxidants such as ascorbic acid or glutathione have also been tested in some systems, although their effectiveness is species- and context-dependent. Overall, no single intervention is universally effective; successful control usually requires balancing medium composition, hormonal regime, gel properties, and physical culture environment.

### 3.7. Genotype Dependence and Protocol Variability

Even after the barriers above are addressed, woody-species tissue culture remains characterized by pervasive genotype dependence: protocols optimized for one cultivar of one species frequently perform poorly when applied to another cultivar of the same species. In *Citrus*, transformation efficiency of epicotyl explants depends strongly on cultivar: above 40% for ‘Carrizo’ citrange and ‘Duncan’ grapefruit, 25% for ‘Hamlin’ sweet orange, and below 10% for Mexican lime and tangerines [101,102,103]; in *Malus*, shoot formation from leaf-derived callus ranged from 8% to 75% among nine apple genotypes [104], and regeneration competence differs markedly among cultivars more generally [105,106,107]; and in *Vitis*, only 24 of 59 genotypes tested produced embryogenic callus from anthers, and the responsive genotypes depended on the growth regulator combination used [108]. This pattern is consistent across many economically important woody genera and represents one of the principal limitations to protocol transferability between laboratories and breeding programs [109].

The biological basis of genotype dependence is multifactorial. Cultivar-specific differences in endogenous plant growth regulator balance, carbohydrate metabolism, secondary metabolite accumulation, ploidy level, and chromatin state can all contribute to variation in in vitro response. Genotype dependence is also frequently confounded with juvenility and maturity effects: elite cultivars are often vegetatively propagated mature genotypes, whereas experimental systems frequently rely on juvenile seedlings or rejuvenated material. As a result, apparent genotype effects may partly reflect differences in physiological age rather than purely genetic variation. The practical consequence is that media formulations and plant growth regulator regimes optimized for one genotype usually cannot be transferred to another without re-optimization. Empirical adjustment of basal salts, carbon sources, hormone concentrations, gelling systems, and culture conditions therefore remain necessary in most woody-species protocols.

Two broad strategies have emerged to address this limitation. First, empirical re-optimization remains the dominant approach, typically involving factorial testing of basal media, plant growth regulator combinations, and culture conditions, increasingly supported by statistical experimental design and machine-learning-assisted optimization, as discussed further in Section 7. Second, genotype-independent regeneration strategies are being developed using morphogenic regulators such as BABY BOOM, WUSCHEL2, and GRF–GIF chimeras, which aim to bypass endogenous regenerative constraints by directly inducing embryogenic or meristematic competence [65,91]. Whether such approaches can achieve robust, scalable genotype independence in woody species remains an open question, but they represent a conceptual shift from genotype-specific protocol tuning toward engineered developmental competence.

The barriers described above interact, as outlined in Section 3.1, which is the main reason why woody-species protocols are difficult to standardize and why media formulation for woody plants has remained more empirical than for many herbaceous systems. The woody-targeted basal media described in the next section each represent a particular set of compromises among these competing demands, optimized for the species, tissue type, and developmental pathway for which they were originally developed.

## 4. Commonly Used Media for Woody Plant Tissue Culture

The basal media described in this section represent practical chemical solutions developed over several decades to address the biological constraints outlined in Section 3. Each formulation can be understood as a compromise among competing physiological demands: supplying sufficient nutrients to support growth without inducing salt or ammonium stress, maintaining ionic strength adequate for cell expansion while minimizing hyperhydricity, and meeting the requirements of perennial woody tissues without exacerbating phenolic exudation or metabolic stress.

We begin with two general-purpose formulations, Murashige and Skoog medium and Gamborg’s B5 medium, which, despite their limitations for many woody species, remain widely used reference media and benchmarks for comparison. We then examine woody-targeted formulations developed in response to limitations of these early media, including Quoirin and Lepoivre medium, Woody Plant Medium, Anderson’s medium, Driver and Kuniyuki Walnut medium, and selected species-specific formulations for olive, conifers, grapevine, rose, and other woody taxa. Compositional differences among these media are summarized in Table 1 (molar equivalents, including total nitrogen, the NO_3_^−^:NH_4_^+^ ratio, and the main cations and anions, are given in Supplementary Table S1), and representative applications are presented in Table 2.

Concentrations are reported in mg/L unless otherwise noted. Values are shown according to the salt forms listed in the table and should be interpreted as formulation equivalents rather than direct elemental concentrations. “-“ indicates that the component is not included in that formulation; “var.” indicates variation among reported or commercial formulations. Molar equivalents of the macronutrient and micronutrient ions are given in Supplementary Table S1.

### 4.1. Murashige and Skoog (MS) Medium

Murashige and Skoog medium, commonly referred to as MS medium, is the most widely used basal medium in plant tissue culture and remains a common starting point for protocol development across diverse plant species [5]. The high ionic strength in MS medium supports rapid cell division and shoot proliferation in many herbaceous systems and underpins its widespread adoption in plant biotechnology [5,22].

For woody species, however, MS medium is often used in modified form rather than at full strength. Half-strength MS, in which macro- and micronutrients are reduced while organic components and plant growth regulators are typically maintained, is widely used during rooting or later culture stages. Reduced ionic strength and nitrogen availability are thought to favor root induction by shifting developmental balance away from shoot proliferation. Modifications that reduce ammonium concentration, including partial or complete removal of ammonium nitrate, are also common, particularly for ammonium-sensitive taxa such as conifers and ericaceous species [22,23]. In addition, substitution of the original MS vitamin formulation with Gamborg’s B5 vitamins is frequently employed and can improve culture vigor or callus quality in some systems.

Despite these modifications, full-strength MS medium is often suboptimal for woody plant culture. Comparative studies have shown that MS-based media can result in reduced regeneration, increased physiological disorders, or poor rooting relative to woody-targeted formulations such as WPM, QL, Anderson’s medium, or DKW, although the magnitude and direction of these effects are strongly species- and genotype-dependent [22,23].

The Nitsch and Nitsch (1969) vitamin mixture is a classical formulation widely used in woody plant micropropagation and tissue culture [158]. It provides a more comprehensive vitamin profile than MS medium and can be used for woody plants that show slow growth, low rooting percentages, or browning issues with standard MS medium.

### 4.2. Gamborg’s B5 Medium

Gamborg’s B5 medium was originally developed for soybean (*Glycine max*) cell suspension cultures and was later adapted for root cultures, protoplast systems, and somatic embryogenesis [49]. Compared with MS medium, B5 contains substantially lower ammonium, a modified vitamin composition (including elevated thiamine, nicotinic acid, and pyridoxine), and a somewhat reduced overall ionic strength.

Although B5 is not a woody-targeted formulation, its reduced ammonium content makes it useful in systems where high ammonium induces physiological stress or inhibits growth. In woody plant tissue culture, B5 is used less frequently than MS or specialized woody media but has found applications in cell suspension cultures, protoplast work, and embryogenic systems, including *Vitis vinifera*, where lower ammonium levels may support embryogenic competence and cell viability. More broadly, the B5 vitamin formulation has had lasting influence: many contemporary protocols combine MS, DKW, or other basal salts with B5 vitamins, reflecting the beneficial effects of enhanced vitamin supply on cellular metabolism.

The origin of B5 in herbaceous cell culture systems, rather than woody tissues, explains its limited direct applicability to woody plant micropropagation. Nevertheless, its compositional features, particularly reduced ammonium and enhanced vitamin supply, have informed the design of later media and hybrid formulations.

### 4.3. Quoirin and Lepoivre (QL) Medium

The Quoirin and Lepoivre (QL) medium, commonly referred to as QL medium, was among the first basal formulations developed specifically to address limitations of MS medium for woody plant culture [24]. Developed for *Prunus* meristem and shoot culture, QL emerged from observations that MS-based systems often produced poor shoot proliferation, chlorosis, or physiological disorders in woody perennials. Through adjustment of mineral composition, Quoirin and Lepoivre reduced total nitrogen and modified nitrogen form, particularly the balance between ammonium and nitrate [24].

QL differs from MS primarily in its reduced total nitrogen and modified nitrogen source. Ammonium nitrate is substantially reduced relative to MS, while calcium nitrate serves as a major nitrogen source, increasing calcium availability and shifting the ammonium:nitrate balance toward nitrate. In molar terms (Supplementary Table S1), QL supplies roughly half the total nitrogen of MS, of which NH_4_^+^ is only about 14% (versus 34% in MS). The increase in calcium is small and depends on the hydration state assumed for calcium nitrate in different sources (≈2.5–3.5 mmol/L versus 3.0 mmol/L in MS), so QL should not be regarded as a high-calcium medium. The formulation also reduces chloride by replacing calcium chloride with calcium nitrate. Micronutrient concentrations are largely similar to those of MS medium, although manganese is substantially reduced [24]. A full compositional comparison with other media is provided in Table 1.

QL has remained most closely associated with *Prunus*, where it performs as well or better than MS medium for meristem culture and shoot proliferation in several species, including *P. avium*, *P. persica*, *P. armeniaca*, and *P. dulcis* [24,115]. More recent studies have used QL as a baseline for further optimization; for example, data-driven refinement of macronutrient composition improved proliferation in hybrid *Prunus* rootstocks, illustrating its continued relevance as a starting formulation [159,160].

Beyond *Prunus*, QL has been applied to other members of the Rosaceae. In rose (*Rosa hybrida*), QL-based media have supported shoot multiplication, although chlorosis has been reported under standard iron–EDTA (FeEDTA), likely reflecting limited iron availability; substitution with ethylenediamine-N,N′-bis(2-hydroxyphenylacetic acid) iron (FeEDDHA) can alleviate this issue [32,147]. In pear (*Pyrus communis*), QL has supported shoot proliferation and adventitious regeneration, although in some cases DKW or modified formulations produce more robust shoots, indicating that optimal media remain species- and genotype-specific [113].

For QL, nitrogen form and total salt concentration appear to be more important than the reduced chloride content sometimes cited as advantageous [24].

### 4.4. Lloyd and McCown’s Woody Plant Medium (WPM)

Relative to MS, WPM is characterized by reduced total ionic strength and a modified nitrogen regime. On a molar basis (Supplementary Table S1), WPM supplies about one quarter of the total nitrogen of MS (14.7 versus 60.0 mmol/L) but keeps the same NO_3_^−^:NH_4_^+^ ratio (≈1.9), so the change lies in the overall nitrogen load rather than in the form of nitrogen supplied. Calcium (3.0 mmol/L) and magnesium (1.5 mmol/L) are equal to those in MS, so their share of the total ionic load rises only because potassium and nitrate are lower. Sulfate, supplied mainly as K_2_SO_4_, is about four times higher than in MS (7.5 versus 1.7 mmol/L), while chloride is lower (1.3 versus 6.0 mmol/L). Micronutrients and vitamins are largely retained from MS with minor adjustments. Full compositional details are provided in Table 1.

WPM has been widely adopted across diverse woody taxa, including ericaceous shrubs such as *Rhododendron* and *Vaccinium*, fruit trees such as *Malus*, *Prunus*, and *Pyrus*, forest species such as *Populus*, *Quercus*, and *Eucalyptus*, and selected nut crops. Its success is generally attributed to its reduced ammonium content and lower ionic strength, which can mitigate physiological disorders such as hyperhydricity and ammonium sensitivity. As a result, WPM is often used as a starting medium for woody species lacking genus-specific protocols [23,25].

Despite its broad applicability, WPM is not universally optimal. In apple (*Malus × domestica*), comparative studies have shown that regeneration and proliferation responses are strongly genotype-dependent, with MS, QL, or DKW outperforming WPM in some cultivars or developmental stages [106,107]. In *Vaccinium*, WPM is widely used but frequently modified, and in some cases Anderson’s medium provides superior performance for acidophilic genotypes. In nut crops such as walnuts, DKW is generally preferred, reflecting species-specific nutritional demands. These examples underscore that WPM should be regarded as a broadly useful baseline rather than a universal solution.

Modification of WPM is common and reflects ongoing empirical optimization. Frequently reported adjustments include altered ammonium:nitrate ratios, incorporation of B5 vitamins, and replacement of FeEDTA with FeEDDHA to address iron deficiency and chlorosis in sensitive species. As with QL, these modifications highlight that basal media function as adaptable frameworks rather than fixed formulations.

WPM has become a major reference medium for woody plant tissue culture and a common baseline against which classical formulations and emerging data-driven optimization approaches are evaluated.

### 4.5. Anderson’s Rhododendron Medium

Anderson’s medium was developed to address poor performance of MS-based formulations in *Rhododendron* and related ericaceous species adapted to acidic, low-nutrient environments [26]. First introduced by Anderson and later more fully characterized, the medium was derived from systematic modification of MS salts to reduce ionic stress and better match the nutritional ecology of acidophilic woody shrubs [26].

Relative to MS, Anderson’s medium is defined by a substantial reduction in total nitrogen and overall ionic strength. These changes produce a medium with lower electrical conductivity, consistent with the low-fertility, acidic soils typical of many ericaceous habitats. In molar terms (Supplementary Table S1), total nitrogen (14.7 mmol/L) and potassium (4.8 mmol/L) are each about one quarter of the MS values (60.0 and 20.1 mmol/L), the NO_3_^−^:NH_4_^+^ ratio is unchanged (~1.9), and calcium and magnesium equal those of MS; the reduction in ionic strength (28 versus 56 mmol/L) therefore reflects mainly lower nitrogen and potassium. In contrast to QL and WPM, Anderson’s medium also increases iron concentration relative to MS, reflecting the sensitivity of *Rhododendron* and related taxa to iron deficiency under standard FeEDTA conditions [26]. Micronutrients are otherwise similar to MS with minor adjustments. Full composition is provided in Table 1.

In *Rhododendron*, it remains an important medium for shoot multiplication and organogenesis, typically used with cytokinin-auxin combinations such as 2-isopentenyladenine and indole-3-acetic acid (IAA) [26]. However, its performance is not universal; comparative studies have shown that WPM can equal or exceed Anderson’s medium in some cultivars, indicating that optimal medium choice remains genotype-dependent [150].

In *Vaccinium*, Anderson’s medium has historically been one of the dominant formulations for micropropagation and regeneration, particularly in highbush and rabbiteye blueberry [52]. Its reduced ionic strength and elevated iron content are generally well suited to these systems, although responses are species- and cultivar-specific. For example, optimized cytokinin–auxin combinations on Anderson’s medium have supported axillary shoot proliferation in highbush and rabbiteye cultivars [52]. In contrast, studies in *Vaccinium meridionale* have reported oxidative stress symptoms and poor shoot quality on Anderson’s medium, highlighting limitations outside its core target group [128].

In *Rubus*, Anderson’s medium has been used for micropropagation and adventitious shoot regeneration, often in modified forms incorporating cytokinins such as TDZ [51,131]. However, MS-based media can produce higher multiplication or regeneration rates in some raspberry cultivars, reinforcing the need for empirical comparison among candidate media [130].

Anderson’s medium and WPM represent parallel solutions to similar biological constraints: both reduce salt stress relative to MS, but they differ in degree of nutrient reduction and iron provision. Increased iron concentration in Anderson’s medium can partially mitigate chlorosis, although further modification, such as FeEDDHA substitution, may still be required in some systems. Economou and Read medium, developed by A.S. Economou and Paul E. Read in 1984/1986, represents a specialized plant tissue culture formulation frequently used alongside Anderson’s *Rhododendron* Medium for micropropagation, organogenesis, and in vitro culture of ericaceous plants including *Rhododendron* and azaleas [161]. These complementary formulations were specifically designed to address the unique nutritional and physiological requirements of acid-loving woody shrubs, offering advantages over generalized media in promoting shoot organogenesis and rooting efficiency.

### 4.6. Driver and Kuniyuki Walnut (DKW) Medium

The Driver and Kuniyuki Walnut (DKW) medium, commonly referred to as DKW medium, was developed for in vitro propagation of Paradox walnut rootstock (*Juglans hindsii* × *J. regia*), a genotype that performed poorly on MS, WPM, and other available formulations [27]. In contrast to QL, WPM, and Anderson’s medium, which reduce total ionic strength relative to MS, DKW represents an alternative strategy: retaining total salt levels comparable to or higher than those of MS while substantially increasing specific mineral components, most notably calcium. In molar terms (Supplementary Table S1), DKW is not a low-salt medium: its macronutrient ionic strength (~87 mmol/L) is about 1.5 times that of MS (~56 mmol/L), reflecting about three times as much calcium (9.4 versus 3.0 mmol/L), twice as much magnesium (3.0 versus 1.5 mmol/L), and about seven times as much sulfate (12.2 versus 1.7 mmol/L), whereas total nitrogen (52.2 versus 60.0 mmol/L) is only modestly lower.

Relative to MS, DKW is characterized by elevated calcium, magnesium, sulfate, and selected micronutrients, with nitrogen moderately reduced but remaining higher than in WPM or Anderson’s medium [27]. Calcium is supplied through both calcium nitrate and calcium chloride, resulting in high calcium availability. Sulfate is increased through potassium sulfate and magnesium sulfate, while copper is also elevated relative to MS. The formulation is commonly used with modified vitamin compositions, and many protocols employ gellan gum rather than agar, consistent with the original walnut system [121]. Full compositional details are provided in Table 1.

The design of DKW reflects nutritional requirements observed in walnut and related woody species. Increased calcium availability is widely associated with improved shoot development and reduced incidence of shoot-tip necrosis, a common disorder in woody plant culture [27,31]. Elevated sulfate and magnesium may also contribute to improved growth, although their specific roles are less well resolved and likely interact with overall nutrient balance. Compared with MS, the moderate reduction in ammonium may reduce physiological stress while maintaining sufficient nitrogen to support active growth.

DKW remains a standard medium for *Juglans* species, where it supports both micropropagation and transformation systems [27,121]. Its use has extended to other nut crops and woody species with similar physiological constraints. In hazelnut (*Corylus avellana*), DKW has frequently outperformed MS and WPM in many cultivars and has served as the basis for extensive optimization studies using response surface methodology and machine-learning approaches [123,162]. These studies highlight the importance of nitrogen form, calcium concentration, and micronutrient balance in determining culture performance. In American chestnut (*Castanea dentata*), DKW medium supported better shoot elongation and proliferation than MS or WPM, with the highest shoot multiplication obtained on DKW supplemented with 2.2 µM BAP and 1.0 µM GA_3_ [163].

Three points emerge from the design of DKW. First, DKW demonstrates that reducing total ionic strength is not the only effective strategy for woody plant media; targeted increases in specific nutrients, particularly calcium, can address key physiological limitations. Second, DKW is especially useful in species prone to shoot-tip necrosis or with high calcium demand, and it often complements rather than replaces lower-salt media such as WPM or Anderson’s medium. Finally, DKW has become an important platform for quantitative media optimization studies, particularly in hazelnut, and continues to inform data-driven approaches to woody plant tissue culture.

### 4.7. Other Species-Specific Woody-Targeted Media

Beyond the major formulations described above, several additional media have been developed for specific woody taxa or for particular stages of in vitro culture. Although these formulations are less broadly applicable than QL, WPM, Anderson’s medium, or DKW, they often provide superior performance within the systems for which they were designed. Representative examples include media for olive, conifers, grapevine, rose, and other woody ornamentals.

Rugini Olive Medium. Rugini’s Olive Medium was developed for *Olea europaea*, a species that often performs poorly on MS- or WPM-based systems [28]. The medium is enriched in calcium, magnesium, sulfur, and selected micronutrients and incorporates additional vitamins such as biotin and folic acid. It also differs in carbon source, with mannitol commonly used in place of sucrose. Zeatin is typically an effective cytokinin for olive shoot proliferation, distinguishing olive from many woody systems where BA or 2iP is more commonly used. Rugini Olive Medium remains a major basal formulation for olive micropropagation and regeneration [28,164].

Litvay and DCR media. Litvay medium and DCR medium are two important basal formulations used in conifer tissue culture and somatic embryogenesis [29,30]. Litvay medium was derived from analyses of developing conifer seed tissues and is widely used, often in modified or reduced-strength form, for embryogenic culture initiation and maintenance in *Pinus*, *Picea*, and related genera [29]. DCR medium, developed by Gupta and Durzan, has also become a standard formulation for conifer embryogenic systems [30]. Comparative studies generally indicate that neither medium is universally optimal; performance varies by species, genotype, explant type, and culture stage.

Chee and Pool C2D medium. C2D medium was developed for *Vitis* micropropagation and represents a modification of MS tailored to grapevine physiology [119]. Adjustments to mineral and micronutrient composition can improve shoot proliferation and culture stability relative to standard MS in some grape cultivars. C2D remains useful for shoot multiplication and rooting in *Vitis vinifera* and related species, although MS-based formulations are often retained for somatic embryogenesis or transformation systems depending on genotype and explant source.

Hasegawa rose media. Hasegawa developed stage-specific initiation and rooting media for *Rosa* that remain influential in rose micropropagation [148,165]. These formulations, derived from MS but modified in nitrogen balance and plant growth regulator composition, illustrate the importance of stage-specific optimization. Their broader significance lies in demonstrating that distinct media for initiation, multiplication, and rooting can outperform single all-purpose formulations, a principle now widely adopted across woody plant tissue culture.

Other formulations. Additional media have been developed for specialized applications, including formulations for woody ornamentals and forestry species [154]. Numerous modified MS-based and hybrid formulations also exist, reflecting incremental optimization for particular species, genotypes, or production systems. Although these media are less widely used than WPM, QL, Anderson’s medium, or DKW, they underscore the continued importance of empirical refinement in woody plant tissue culture.

The collective lesson from these specialized formulations is consistent with the broader theme of this section: woody-species tissue culture media constitute a portfolio of solutions, each optimized for specific physiological and developmental constraints. This diversity reflects the biological heterogeneity of woody plants rather than inconsistency in the field. In practice, basal-medium selection should be guided by taxonomic and physiological similarity to previously studied systems-for example, Rugini Olive Medium for olive, Litvay or DCR media for conifers, C2D for grapevine, and Hasegawa or QL-based systems for rose. Nevertheless, empirical comparison of candidate media remains essential when establishing protocols for new species, cultivars, explant sources, or regeneration pathways.

## 5. Media Components and Their Roles in Woody-Species Culture

The basal media described in Section 4 are not merely chemical inventories; they are integrated formulations in which mineral salts, carbon sources, vitamins, plant growth regulators, organic additives, gelling agents, and pH collectively determine tissue survival, growth, and morphogenic competence. This section examines these components individually, with emphasis on how they are configured in woody-targeted media and why woody species often respond differently from herbaceous model systems. Many classical tissue culture principles were developed using tobacco, carrot, and other herbaceous models, whereas trees and shrubs impose additional physiological constraints, including sensitivity to high ammonium and high total salt strength, strong calcium requirements in lignifying shoots, iron-limited chlorosis during prolonged subculture, cytokinin-associated hyperhydricity or shoot-tip necrosis, phenolic oxidation, slow growth, and genotype-dependent recalcitrance.

### 5.1. Macronutrients

Macronutrients, including nitrogen, phosphorus, potassium, calcium, magnesium, and sulfur, provide the major inorganic ions required for protein synthesis, nucleic acid metabolism, membrane function, cell-wall formation, and osmotic balance. Macronutrient proportions in culture media differ markedly from those used in horticultural fertilizers: on an elemental mass basis, MS medium has an N:P:K ratio of approximately 22:1:20, and the formulations discussed here vary widely around this pattern (Supplementary Table S1), so fertilizer-derived ratios such as 3:1:2 should not be assumed to be optimal in vitro. Although carbon, hydrogen, and oxygen account for most plant tissue dry mass, macronutrient salts dominate the ionic composition and electrical conductivity of culture media and are therefore major determinants of in vitro growth behavior [2,21,22]. For woody species, three macronutrient features are especially consequential: total nitrogen and the NH_4_^+^:NO_3_^−^ balance, calcium supply, and overall ionic strength.

Nitrogen forms and ratios. Most plant tissue culture media supply nitrogen as a combination of nitrate and ammonium. Plant cells can grow with nitrate as a major nitrogen source, but many tissues benefit from some reduced nitrogen because ammonium or amino acids are metabolically less expensive to assimilate than nitrate, which must first be reduced before incorporation into amino acids [2,21]. However, excessive ammonium can inhibit growth, and many woody species are sensitive to the high ammonium levels present in full-strength MS medium [23,47]. Woody-targeted formulations such as WPM, Anderson’s medium, and QL medium therefore reduce ammonium concentration relative to MS medium while maintaining nitrate as the dominant nitrogen source [24,25,26]. Supplementary Table S1 shows that, for WPM and Anderson’s medium, this is achieved mainly by lowering total nitrogen to about one quarter of the MS value: the NO_3_^−^:NH_4_^+^ molar ratio is essentially unchanged (≈1.9, as in MS) and is higher mainly in QL (≈6), Rugini OM (≈4), and B5 (≈12), and moderately in DCR (≈2.6). The biochemical basis of ammonium toxicity includes cytoplasmic acidification, disruption of mitochondrial metabolism, and depletion of carbon skeletons required for amino acid synthesis [47]. The practical consequence is that the NH_4_^+^:NO_3_^−^ ratio can influence both growth rate and morphogenic outcome. High-ammonium or auxin-rich media may support callus formation in some systems, whereas lower-ammonium media are often more favorable for shoot proliferation, shoot quality, and rooting in woody species. Importantly, ammonium sensitivity does not mean that woody species universally prefer nitrate alone; rather, many woody taxa require ammonium at physiologically tolerable concentrations.

Calcium and shoot-tip necrosis. Calcium is required for cell-wall stability, membrane integrity, signaling, and meristem function. In woody species, calcium is particularly important because lignifying shoots and actively growing apices are prone to localized calcium deficiency under in vitro conditions. Shoot-tip necrosis, a recurring disorder in *Juglans*, *Pistacia*, *Pyrus*, *Cydonia*, and other woody crops, has been associated with insufficient calcium delivery to rapidly growing shoot tips [31]. Because calcium moves primarily through the transpiration stream, in vitro shoots maintained in high-humidity vessels with low transpiration are especially vulnerable to calcium limitation at the apical meristem. DKW medium addresses this constraint by providing elevated calcium relative to many other basal media, which likely contributes to its effectiveness in walnut and other shoot-tip-necrosis-prone species [27]. Calcium availability also interacts with the gelling system, because agar or gellan gum can influence water availability, transpiration, and divalent cation mobility [31].

Ionic strength and total macronutrient load. Full-strength MS medium has high total ionic strength, which supports rapid growth in many herbaceous systems but can be excessive for salt-sensitive woody taxa [22,23]. Woody-targeted media represent different strategies for managing ionic strength. WPM, QL, and Anderson’s medium reduce total salt concentration relative to MS, whereas DKW retains a total salt load comparable to or higher than that of MS but increases selected nutrients, especially calcium and magnesium [24,25,26,27]. Ionic-strength-sensitive species, particularly ericaceous shrubs and some conifers, often perform better on lower-salt formulations, whereas more salt-tolerant woody taxa may tolerate MS but still benefit from modified nitrogen, calcium, or micronutrient profiles. Thus, total ionic strength should be considered together with nitrogen form, calcium supply, and genotype-specific tolerance.

### 5.2. Micronutrients

Micronutrients are required at trace concentrations but are essential as enzyme cofactors, in chlorophyll biosynthesis, redox balance, and hormone-related metabolism. Most woody-targeted media retain the standard micronutrient set found in MS medium, including boron, manganese, zinc, iodine, molybdenum, copper, cobalt, and iron, but modify selected elements to address species-specific deficiencies or physiological disorders. Two aspects are especially important in woody species: iron availability and the requirements for copper, zinc, and related trace elements.

Iron availability and the FeEDTA limitation. Iron deficiency in tissue culture commonly appears as interveinal chlorosis, reduced shoot multiplication, and, in severe cases, shoot decline [166]. Most basal media supply iron by combining FeSO_4_·7H_2_O with Na_2_EDTA·2H_2_O, which forms an FeEDTA chelate in solution. Although FeEDTA provides adequate iron availability in many standard tissue culture media, its effectiveness can decline during prolonged culture because of pH-dependent chelate stability, interactions with phosphate, light sensitivity, and precipitation or degradation during medium preparation and storage. In woody species, these limitations can become especially important in systems prone to chlorosis, including acidophilic or iron-sensitive taxa and cultures maintained through repeated subculture. Anderson’s medium partly addresses this issue by increasing iron concentration, reflecting the sensitivity of *Rhododendron* and related ericaceous species to iron limitation [26]. In rose and other chlorosis-prone woody species, substitution of FeEDTA with the more stable chelate FeEDDHA has been shown to improve iron availability, shoot greenness, and culture performance [32]. Thus, iron form can be as important as iron concentration, particularly for acidophilic, iron-sensitive, or long-term subcultured woody tissues.

Copper, zinc, and other trace elements. Several woody-targeted media increase copper or zinc relative to MS. DKW and WPM contain elevated copper compared with MS, while Rugini Olive Medium increases selected micronutrients, including zinc, consistent with the mineral requirements of olive tissues [25,27,28]. Litvay and DCR media also differ from MS in trace-element balance, reflecting their development for conifer systems [29,30]. These adjustments are not merely minor compositional differences; copper contributes to oxidative metabolism and lignification-related enzymes, while zinc functions in transcriptional regulation, protein structure, and auxin metabolism. DKW also includes nickel, a micronutrient required for urease activity and nitrogen metabolism [27]. The practical consequence is that switching basal media changes a coordinated package of micronutrients, not simply macronutrient concentrations. Direct substitution of one woody-targeted medium for another therefore usually requires empirical re-optimization.

### 5.3. Carbon Sources

Plant cells in culture often have limited photosynthetic capacity and depend on the medium for both carbon skeletons and metabolic energy. The carbon source influences growth rate, osmotic potential, tissue quality, and developmental fate [2]. In woody species, sucrose remains the default carbon source, but maltose, mannitol, glucose, and sorbitol are used in selected systems.

Sucrose. Sucrose at 20–30 g/L is the most common carbon source in woody-species protocols. It is inexpensive, readily metabolized, stable during autoclaving, and provides an appropriate osmotic environment for shoot multiplication and rooting. WPM commonly uses a lower sucrose concentration (20 g/L) than MS, DKW, QL, and DCR (30 g/L), consistent with its lower-salt design and its use in salt- or osmotic-sensitive woody species [25]. Reduced sucrose concentrations are often used during rooting, where lower osmotic potential may favor the developmental transition from shoot proliferation to root induction [2].

Maltose and somatic embryogenesis. Maltose is frequently used in somatic embryogenesis protocols, especially in conifers, because its slower hydrolysis can provide a more gradual carbon supply and reduce osmotic shock during embryo maturation. In conifer somatic embryogenesis systems using Litvay or DCR-based media, maltose is often substituted for sucrose during maturation or germination stages [29,30]. Although maltose is more expensive than sucrose, the improvement in embryo quality or synchronization can justify its use in somatic embryogenesis pipelines.

Mannitol in olive culture. Rugini Olive Medium is distinctive in its frequent use of mannitol as a carbon or osmoticum component for *Olea europaea* culture [28]. This reflects the physiology of olives and the need to manage growth, osmotic balance, and browning in a phenolic-rich woody species. The mannitol-based approach is largely associated with olive and selected Mediterranean woody species rather than being a general replacement for sucrose. Mannitol functions primarily as an osmoticum and stress-inducing agent rather than a nutrient source in woody plant tissue culture. Beyond its role as an osmoticum, mannitol can impose osmotic stress, one of several stress treatments reported to trigger cellular reprogramming and promote the induction of somatic embryogenesis [167].

Other carbon sources. Anhydrous glucose can substitute for sucrose in some systems, but it is less commonly used because sucrose generally provides stable and reproducible performance. Fructose is usually less effective than sucrose or glucose. Sorbitol, a major translocated carbohydrate in many Rosaceae, has been tested in apple, pear, and *Prunus*, but results are mixed and it has not displaced sucrose as a standard carbon source. Overall, sucrose remains the default for most woody-species protocols, while maltose is most relevant to somatic embryogenesis and mannitol to olive culture. It should be noted that glucose and fructose, as reducing sugars, are chemically unstable in culture media. Even without autoclaving, they gradually undergo non-enzymatic degradation (isomerization, Maillard reactions with amino acids and other nitrogenous components, and formation of furan derivatives and organic acids) during medium storage and culture. Heat accelerates these reactions, so autoclaving increases degradation substantially [168]. Sucrose itself is partly hydrolyzed to glucose and fructose during autoclaving and by cell-wall invertases during culture. The actual carbohydrate composition of the medium therefore changes over time.

### 5.4. Vitamins

Vitamins function as cofactors in central metabolism, including amino acid biosynthesis, energy metabolism, and one-carbon transfer reactions. Although they constitute a small fraction of the medium by mass, vitamin composition can substantially influence culture vigor, callus quality, and morphogenic competence [2,21].

Standard vitamins: thiamine, nicotinic acid, pyridoxine, and glycine. Thiamine, or vitamin B1, is the most consistently required vitamin in plant tissue culture and functions as a cofactor in thiamine-pyrophosphate-dependent metabolic reactions. MS medium contains relatively low thiamine, whereas Gamborg’s B5 medium contains substantially higher thiamine (10 versus 0.1 mg/L) [5,49]. Many woody-targeted media, including WPM and DKW, use higher thiamine concentrations than standard MS, reflecting the broader influence of B5-type vitamin formulations on woody-species culture [25,27]. Nicotinic acid and pyridoxine are retained in most basal media, while glycine is included in many formulations as a non-essential organic nitrogen supplement that may support growth or somatic embryogenesis in selected systems.

B5 vitamins. Although B5 basal salts are not widely used as the primary medium for woody micropropagation, the B5 vitamin formulation has had a lasting influence. Many protocols combine MS, DKW, or other basal salts with B5 vitamins to improve tissue vigor, callus quality, or regeneration response. This hybrid strategy illustrates an important principle: basal salts and vitamin formulations can be optimized independently, and the best-performing formulation for a given woody species may combine components from different classical media.

Biotin, folic acid, and myo-inositol. Some woody-targeted media include vitamins not present in standard MS. Rugini Olive Medium includes biotin and folic acid, while QL medium includes biotin, reflecting empirical optimization for olive and *Prunus* culture systems [24,28]. Biotin functions as a cofactor for carboxylase enzymes, whereas folic acid supports one-carbon metabolism. Myo-inositol, historically grouped with vitamins, is included in nearly all plant tissue culture media and contributes to cell-wall biosynthesis, membrane signaling, and phosphate metabolism [2]. Thus, switching from MS to a woody-targeted formulation changes not only macro- and micronutrient composition but also the vitamin environment, and these combined effects can influence woody-species culture performance.

### 5.5. Plant Growth Regulators

Plant growth regulators (PGRs) are among the most consequential variables in tissue culture media. The choice of PGR class, specific compound, concentration, and timing determines whether an explant forms callus, shoots, roots, or somatic embryos. In woody species, PGR optimization is often more empirical and more genotype-specific than in herbaceous models, but the underlying principle remains the same: auxins and cytokinins act in balance to direct morphogenesis [6]. Other PGR classes, including gibberellins, abscisic acid, ethylene inhibitors, brassinosteroids, and melatonin, can modulate this central auxin–cytokinin framework.

#### 5.5.1. Auxins

Auxins promote cell division, cell expansion, vascular differentiation, rooting, callus formation, and somatic embryogenesis, depending on the compound and concentration used.

IAA, NAA, and IBA. Indole-3-acetic acid is the principal natural auxin but is relatively unstable in culture media and is rapidly degraded in many tissues. Naphthaleneacetic acid and indole-3-butyric acid are more stable and are widely used in woody-species protocols. IBA is particularly important for in vitro rooting and is commonly used during rooting stages in *Prunus*, *Pyrus*, *Malus*, *Vaccinium*, *Rubus*, and *Rosa* [52,111,118,126,148]. NAA is more frequently used for callus induction or in low concentrations during shoot proliferation in combination with cytokinins.

2,4-D and picloram. 2,4-Dichlorophenoxyacetic acid and picloram are persistent synthetic auxins that strongly induce dedifferentiation, callus formation, and somatic embryogenesis. 2,4-D has historically been the most widely used auxin for somatic embryogenesis induction, especially in conifers. However, auxin type can strongly influence outcome. In *Pinus*, comparative studies have shown that 2,4-D, picloram, and NAA differ in their effects on embryogenic tissue initiation, proliferation, oxidative stress, and plantlet quality [140]. Picloram has also shown strong effects in somatic embryogenesis systems of *Lycium barbarum* and selected palm species [169,170,171]. These findings support testing 2,4-D, picloram, and NAA in parallel when developing new woody-species somatic embryogenesis systems rather than defaulting to 2,4-D alone.

#### 5.5.2. Cytokinins

Cytokinins promote cell division and, when supplied in excess relative to auxin, stimulate shoot proliferation and adventitious shoot formation. The cytokinin landscape in woody-species tissue culture has expanded beyond BAP to include zeatin, 2iP, TDZ, and aromatic cytokinins such as meta-topolin.

BAP and adenine-derived cytokinins. 6-Benzylaminopurine has been the most widely used cytokinin in plant tissue culture because it is inexpensive, stable, and effective across many species. In woody protocols, BAP is commonly used for shoot proliferation and adventitious shoot induction. Kinetin and 2-isopentenyladenine are alternative adenine-derived cytokinins. 2iP is commonly used in *Rhododendron* culture on Anderson’s medium, while zeatin is particularly important in olive and some *Vaccinium* protocols [26,28,52]. Zeatin is more expensive than BAP but may produce superior shoot quality in species where BAP causes physiological disorders.

TDZ. Thidiazuron is a synthetic phenylurea cytokinin with strong cytokinin-like activity and, in some contexts, auxin-like effects. TDZ can induce high shoot regeneration rates from recalcitrant explants and is widely used in woody-species adventitious regeneration. However, TDZ can also promote hyperhydricity, shoot-tip necrosis, excessive callus formation, and poor shoot elongation if used at high concentration or for prolonged exposure [20,114]. For this reason, TDZ is often used as a short induction pulse and then replaced with BAP, zeatin, or meta-topolin for shoot elongation and maintenance.

Meta-topolin and aromatic cytokinins. Meta-topolin is an aromatic cytokinin originally identified in poplar leaves and chemically related to BAP [172]. Comparative studies have shown that meta-topolin can improve shoot quality, reduce hyperhydricity, enhance rooting, and reduce shoot-tip necrosis in several woody and ornamental species. In apple, meta-topolin produced more normal shoots than BAP in some genotypes [173]. In *Lagerstroemia speciosa*, meta-topolin improved shoot multiplication and post-transplant performance relative to BAP [153]. Thus, when BAP-based protocols produce hyperhydric, short, or low-quality shoots in woody species, substituting meta-topolin at comparable molar concentrations is a rational optimization step.

#### 5.5.3. Other PGR Classes

Gibberellins. Gibberellic acid is included in some woody-species protocols to promote shoot elongation or overcome dormancy-related growth inhibition. It is used selectively in rose and some woody ornamental protocols but is generally avoided during rooting because gibberellins can inhibit root initiation [148,174].

Abscisic acid. Abscisic acid (ABA) is widely used during the maturation phase of somatic embryogenesis, especially in conifers, where it suppresses precocious germination and supports cotyledon development, desiccation tolerance, and storage reserve accumulation [175]. ABA is therefore most relevant to two-stage somatic embryogenesis systems rather than routine shoot multiplication.

Ethylene inhibitors. Ethylene accumulation can contribute to senescence, tissue browning, and reduced regeneration after wounding or *Agrobacterium* co-cultivation. Silver nitrate and related ethylene inhibitors have therefore been used in some regeneration and transformation systems to reduce ethylene-associated stress responses [14]. Their effectiveness is species- and dose-dependent and should be optimized carefully because silver compounds can also affect development.

Brassinosteroids and melatonin. Brassinosteroids and melatonin have emerged as ancillary regulators that may improve regeneration or stress tolerance in selected woody systems. Their use is not yet standard, but they represent active areas of optimization, especially for transformation pipelines where oxidative stress and wound responses limit regeneration.

#### 5.5.4. Pathway-Dependent PGR Design

The principle that the auxin-to-cytokinin ratio influences morphogenic fate remains foundational, but woody-species protocol development shows that the identity of the auxin and cytokinin, the timing of exposure, and the culture stage are equally important [6]. In this view, exogenous cytokinin acts less as a direct organogenic signal than as a modulator of endogenous auxin distribution, locally promoting or repressing auxin biosynthesis depending on cell context [176] and altering polar auxin transport [177]. Its effect depends on the presence of competent cells able to respond, and competence itself appears to be defined partly by local auxin availability [176]. Different developmental pathways require systematically different PGR profiles.

Callus induction is favored by intermediate-to-high concentrations of persistent auxins such as 2,4-D, NAA, or picloram, often with low cytokinin or no cytokinin. In woody species, callus induction is often easier than subsequent regeneration, and the main bottleneck is usually recovery of organized shoots, roots, or embryos from callus tissue.

Indirect organogenesis requires transfer from an auxin-dominant callus induction medium to a cytokinin-dominant regeneration medium. BAP remains a common starting cytokinin; TDZ may be useful for recalcitrant explants; and meta-topolin is increasingly used when BAP or TDZ causes hyperhydricity or poor shoot quality. Low concentrations of NAA or IBA may support cell division without overriding the cytokinin signal. The disadvantage of indirect organogenesis is that prolonged callus phases can increase somaclonal variation and reduce clonal fidelity.

Direct organogenesis is increasingly preferred for woody-species transformation pipelines because it minimizes the callus phase and can reduce epigenetic or somaclonal variation. Direct organogenesis typically uses lower auxin and higher cytokinin than indirect pathways, often with TDZ, BAP, zeatin, or meta-topolin depending on species and explant type. Nodal segments, juvenile leaf explants, cotyledons, hypocotyls, and immature embryo-derived tissues are commonly used.

Somatic embryogenesis usually requires high concentrations of persistent auxins such as 2,4-D or picloram during induction, followed by substantial auxin reduction or withdrawal during embryo maturation. ABA and osmotic adjustments, often including maltose substitution for sucrose, support maturation and improve embryo quality [140,175]. This two-phase structure distinguishes somatic embryogenesis from most organogenesis protocols and is one of the more demanding aspects of woody-species regeneration.

Rooting is typically induced by transferring shoots to media with reduced or eliminated cytokinin and a single auxin, most commonly IBA. Half-strength MS or WPM is widely used for rooting because reduced ionic strength can favor root initiation [2,25]. For difficult-to-root woody species, brief auxin pulses followed by transfer to auxin-free medium often outperform continuous auxin exposure.

Shoot proliferation from axillary buds and shoot tips differs from adventitious organogenesis because existing meristems are stimulated rather than new ones formed. It requires a cytokinin-dominant medium with little or no auxin, and the main constraint is the cytokinin level at which multiplication is sustained without hyperhydricity, shoot-tip necrosis, or loss of shoot quality over successive subcultures [20]. The requirements of the four main pathways are compared in Table 3.

Concentration, exposure time, and sequence of culture phases. Three aspects of PGR design deserve explicit attention. First, concentration effects are non-linear and compound-specific: the optimum for one auxin or cytokinin cannot be converted to another on a mass basis, and concentrations that promote one response (for example, root initiation) inhibit the next (root elongation). Second, exposure time can matter as much as concentration. TDZ is often used as a short induction pulse because prolonged exposure promotes callus, hyperhydricity, and poor elongation [20,114]; an auxin pulse followed by transfer to auxin-free medium commonly outperforms continuous auxin for rooting; and prolonged exposure to 2,4-D or picloram prevents the progression from induction to embryo maturation [140,175]. Third, regeneration in woody species usually requires a defined sequence of media rather than a single formulation. The regulator that induces competence (an auxin-rich or TDZ-containing medium) often inhibits the following phase of development, so induction, expression, elongation, and rooting are typically carried out on different media. Carry-over of regulators between phases, and the endogenous hormone state of the explant at the time of transfer, can confound the interpretation of single-phase experiments. Because endogenous hormone status differs among genotypes and between juvenile and mature material (Section 3.4 and Section 3.7), concentration, exposure time, and phase duration generally need to be re-optimized for each genotype.

**Figure 4:**
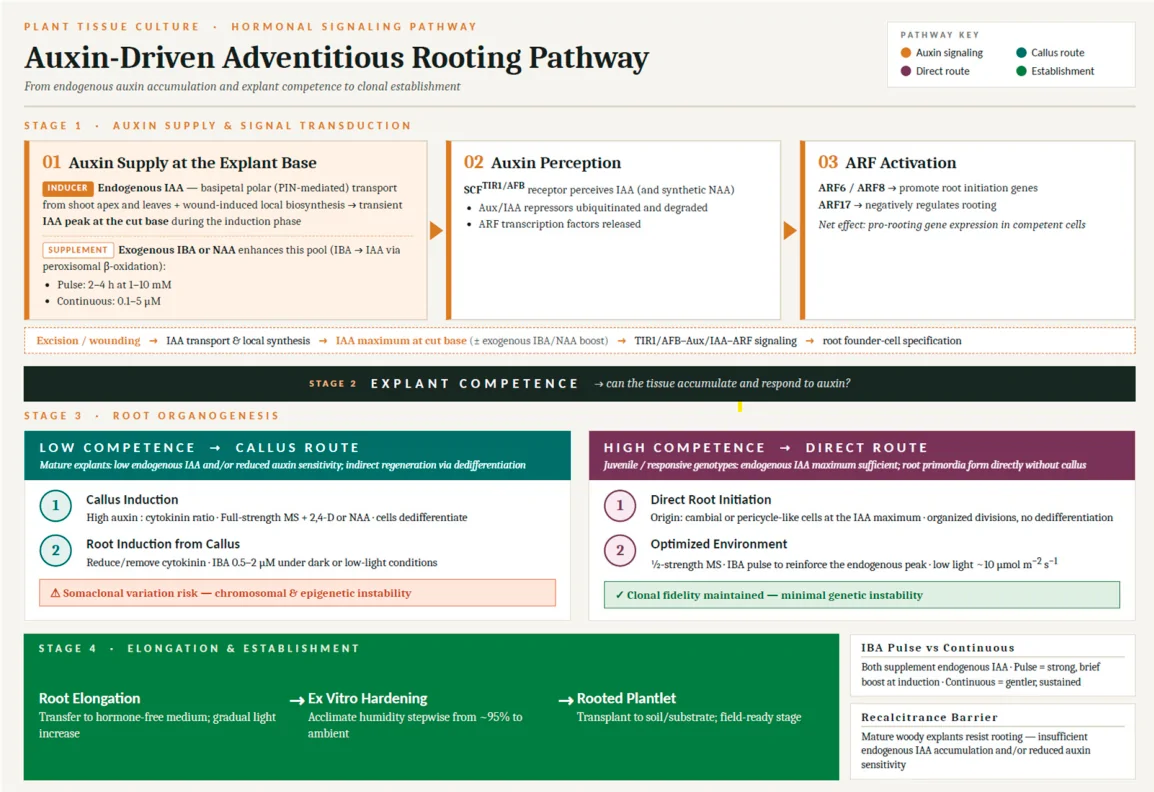
Auxin-driven adventitious rooting in plant tissue culture. Rooting is induced by endogenous indole-3-acetic acid (IAA). After excision, IAA reaches the explant base through PIN-mediated polar transport and wound-induced local biosynthesis, where it forms a transient maximum. Exogenous IBA (converted to IAA by β-oxidation) or NAA, given as a pulse or continuously, adds to this pool. SCF^TIR1/AFB^ perceives the auxin, Aux/IAA repressors are degraded, and ARF6/8 are activated while ARF17 restrains rooting. Explant competence decides the route. Low-competence mature explants regenerate indirectly through callus, which risks somaclonal variation. High-competence juvenile genotypes form root primordia directly from cambial or pericycle-like cells, which preserves clonal fidelity. Rooted shoots are then elongated on hormone-free medium, hardened ex vitro and transplanted.

Overall, woody-species protocols require coordinated PGR decisions across culture stages. Auxin type, cytokinin type, concentration, exposure duration, and the timing of transitions all contribute to morphogenic outcome. The basal media described in Section 4 provide the mineral and vitamin foundation, but the PGR regime must be optimized for each species, genotype, explant source, and developmental pathway. The auxin-related developmental cascade and the distinction between direct and callus-mediated regeneration are summarized in Figure 4. Although exogenous auxins are routinely applied in vitro, the rooting response depends on the endogenous IAA peak that forms at the explant base shortly after excision; applied IBA and NAA enhance this peak rather than replace it.

### 5.6. Organic Additives

Beyond defined macronutrients, micronutrients, vitamins, and PGRs, tissue culture media frequently include organic additives such as coconut water, casein hydrolysate, malt extract, yeast extract, polyamines, glutamine, and activated charcoal. These additives are common in woody-species protocols because they can supply amino acids, organic acids, growth-promoting factors, or adsorptive capacity that defined components alone may not provide.

Potato extract is another example: it provides complex organic compounds, micronutrients, and undefined growth-promoting factors and has been used to support shoot organogenesis and rooting. Peptones, which are enzymatic digests of protein, supply amino acids and peptides as organic nitrogen, and different peptone types differ in nitrogen speciation and osmotic properties.

Coconut water. Coconut water, the liquid endosperm of *Cocos nucifera*, has been used in plant tissue culture since early studies demonstrated its growth-promoting activity [178]. It supplies natural cytokinin, amino acids, sugars, minerals, and other growth-promoting factors. Although sometimes described as an alternative to synthetic PGRs, coconut water is more accurately viewed as a complex natural cytokinin and nutrient source. Its principal limitations are batch-to-batch variability, microbial contamination risk, and difficulty in standardization. For rigorous research protocols, defined cytokinins are generally preferred, although coconut water remains useful in some commercial and recalcitrant systems.

Casein hydrolysate, peptone, and amino acids. Casein hydrolysate and peptone are complex organic nitrogen supplements that provide mixtures of amino acids, peptides, and trace organic nutrients. These additives can support cell suspension growth, somatic embryogenesis, and embryo maturation in selected woody species, especially conifers [175]. However, because their composition can vary among sources and batches, defined amino acids such as glutamine, asparagine, or proline may be preferable when reproducibility is important. Glutamine is included in some woody media and transformation protocols as a reduced nitrogen source that can support tissue recovery after stress, including wounding or *Agrobacterium* co-cultivation. In Rugini Olive Medium, glutamine has been reported as part of organic nitrogen supplementation, although reported concentrations may vary among formulation sources [28].

Polyamines and other complex supplements. Polyamines such as putrescine, spermidine, and spermine have been tested in woody-species regeneration and somatic embryogenesis systems, where they may influence oxidative stress, cell division, and hormone balance. Malt extract and yeast extract were common in older protocols but are now less favored in research settings because their composition is poorly defined. In general, complex organic additives can have substantial effects on woody-species growth and morphogenesis, but defined alternatives are preferable when reproducibility across laboratories or years is required.

Activated charcoal. Activated charcoal is widely used in woody-species tissue culture for two purposes. First, it absorbs phenolic exudates and oxidation products, reducing tissue browning in phenolic-rich species such as olive, walnut, chestnut, mango, and some conifers [64]. Second, it adsorbs inhibitory PGRs or residual compounds, which can be useful during embryo maturation or after high-auxin induction phases. The limitation is that activated charcoal is non-selective and can also remove added PGRs, vitamins, and beneficial organic compounds. Protocols using activated charcoal therefore often require adjustment of PGR and vitamin concentrations. Activated charcoal is supplied from several sources, including coconut-derived, bamboo, bone, and USP-grade hardwood charcoal, which differ in adsorptive capacity, mineral content, and suitability for regulated applications, and therefore in efficacy. Selection of appropriate charcoal type and concentration (0.2–3% *w*/*v*) can improve culture clarity and plantlet quality in recalcitrant woody species, although effects depend on species and charcoal type [64].

### 5.7. Gelling Agents

The gelling agent provides the semi-solid matrix on which many tissue cultures are maintained. It affects physical support, water availability, gas diffusion, nutrient mobility, and ion availability. In woody species, gelling agents can strongly influence hyperhydricity, shoot-tip necrosis, rooting, and overall shoot quality.

Agar. Agar remains the traditional and most widely used gelling agent in plant tissue culture. It is inexpensive, autoclavable, and generally reliable. However, agar can contain variable impurities, bind divalent cations such as calcium, and produce relatively soft gels with high water availability, which may contribute to hyperhydricity in cytokinin-rich media [19,20,31]. Increasing agar concentration can produce firmer gels and reduce hyperhydricity, but overly firm media may reduce explant contact with the medium and slow growth.

Gellan gum. Gellan gum, sold under trade names such as Gelrite or Phytagel, forms clear, firm gels at lower concentrations than agar. It generally contains fewer organic impurities and may provide more consistent gel properties. In several woody systems, gellan-gelled media improve shoot quality or reduce physiological disorders, particularly where calcium availability and hyperhydricity are concerns [27,121]. However, responses are species-specific, and some woody taxa perform better on agar. Testing agar and gellan gum in parallel is therefore a useful step when establishing new protocols.

Liquid culture and temporary immersion. In suspension cultures, somatic embryogenesis systems, and large-scale micropropagation, gelling agents may be eliminated entirely. Temporary immersion systems periodically immerse explants in liquid medium and then drain the medium, combining improved nutrient access with better aeration than continuous liquid culture [179]. These systems can be useful for woody micropropagation and somatic embryogenesis but require careful control of immersion frequency, vessel ventilation, and hyperhydricity.

### 5.8. pH

Although pH is one of the most fundamental variables in plant tissue culture media, it is often underreported or inadequately controlled. Most basal media are adjusted to pH 5.6–5.8 before autoclaving, a range suitable for many plant tissues and compatible with the solubility of most medium components [2]. The commonly reported pH of 5.7 is the value adjusted before autoclaving. Autoclaving usually lowers pH, and during culture the medium pH changes rapidly, often acidifying within the first days [180]. This is driven mainly by ion uptake (NH_4_^+^ uptake acidifies the medium, NO_3_^−^ uptake alkalinizes it) and Fby proton extrusion through the plasma membrane H^+^-ATPase [7]. These changes affect: (i) nutrient availability, including Fe precipitation and chelate stability, phosphate speciation and Ca/Mg phosphate precipitation [181]; (ii) the properties of gelling agents, as agar solidifies poorly below about pH 4.5 and gellan gum gel strength depends on divalent cations and pH [182]; and (iii) auxin uptake and transport, which are pH dependent [183]. For woody species, pH is important because it affects nutrient solubility, iron availability, gel strength, microbial growth, and species-specific physiological responses.

Species-specific pH preferences. While pH 5.6–5.8 is suitable for many woody species, acidophilic taxa may perform better at lower pH. Anderson’s medium for *Rhododendron* was developed for ericaceous shrubs adapted to acidic, low-nutrient soils and is typically used at a lower pH than standard MS-based formulations [26]. *Vaccinium* species may also benefit from acidic media. Conversely, some woody species may perform better at slightly higher pH depending on nutrient availability and culture stage. Thus, pH should be treated as an adjustable variable rather than a fixed default. The pH is usually adjusted before autoclaving with NaOH or KOH to raise it or with HCl to lower it. Autoclaving and pH drift. Medium pH can change during autoclaving and during culture as tissues absorb ions and exude organic acids. The direction and magnitude of pH drift depend on basal salts, sucrose concentration, gelling agent, and buffer capacity [180,183]. Because pH affects iron solubility, gelling behavior, and tissue response, pH should ideally be measured after autoclaving during protocol development, particularly for sensitive woody species. Buffers such as MES can stabilize pH in some systems, but buffer concentration should be optimized because excessive buffering can also alter tissue response. MES (2-(N-morpholino)ethanesulfonic acid) represents an advanced buffering system for plant tissue culture media, offering superior pH stability [183]. MES provides effective buffering near the pH commonly used for plant tissue culture. When MES free acid is partially neutralized or combined with its potassium salt, the resulting conjugate acid–base pair provides buffering within the effective MES range of approximately pH 5.5–6.7, helping minimize pH fluctuations during culture [183]. This combination system is particularly valuable in woody plant micropropagation, where stable pH is critical for nutrient availability, hormone stability, and consistent developmental responses. Besides chemical buffers, the medium pH can be stabilized physiologically. The main tool is adjusting the NH_4_^+^:NO_3_^−^ ratio so that acidifying and alkalinizing uptake balance each other. Other contributors include organic acids (e.g., citrate, malate, succinate), Tris, amino acids or casein hydrolysate, and the carbonic acid/bicarbonate system [2,184].

Gelling agent and pH interactions. Gel formation is influenced by pH and divalent cation availability. Agar generally gels across the pH range used in plant tissue culture, but gel strength can decline at more acidic pH. Gellan gum depends strongly on calcium and magnesium availability and can therefore interact with the mineral composition of the medium. For low-pH media such as Anderson’s medium, gelling agent concentration or type may need adjustment to maintain appropriate firmness and water availability. These interactions reinforce the broader theme of this section: medium components cannot be optimized independently, because salts, pH, gelling agents, PGRs, and explant physiology interact to determine woody-species culture performance.

### 5.9. A Framework for Selecting and Modifying a Basal Medium

**Figure 5:**
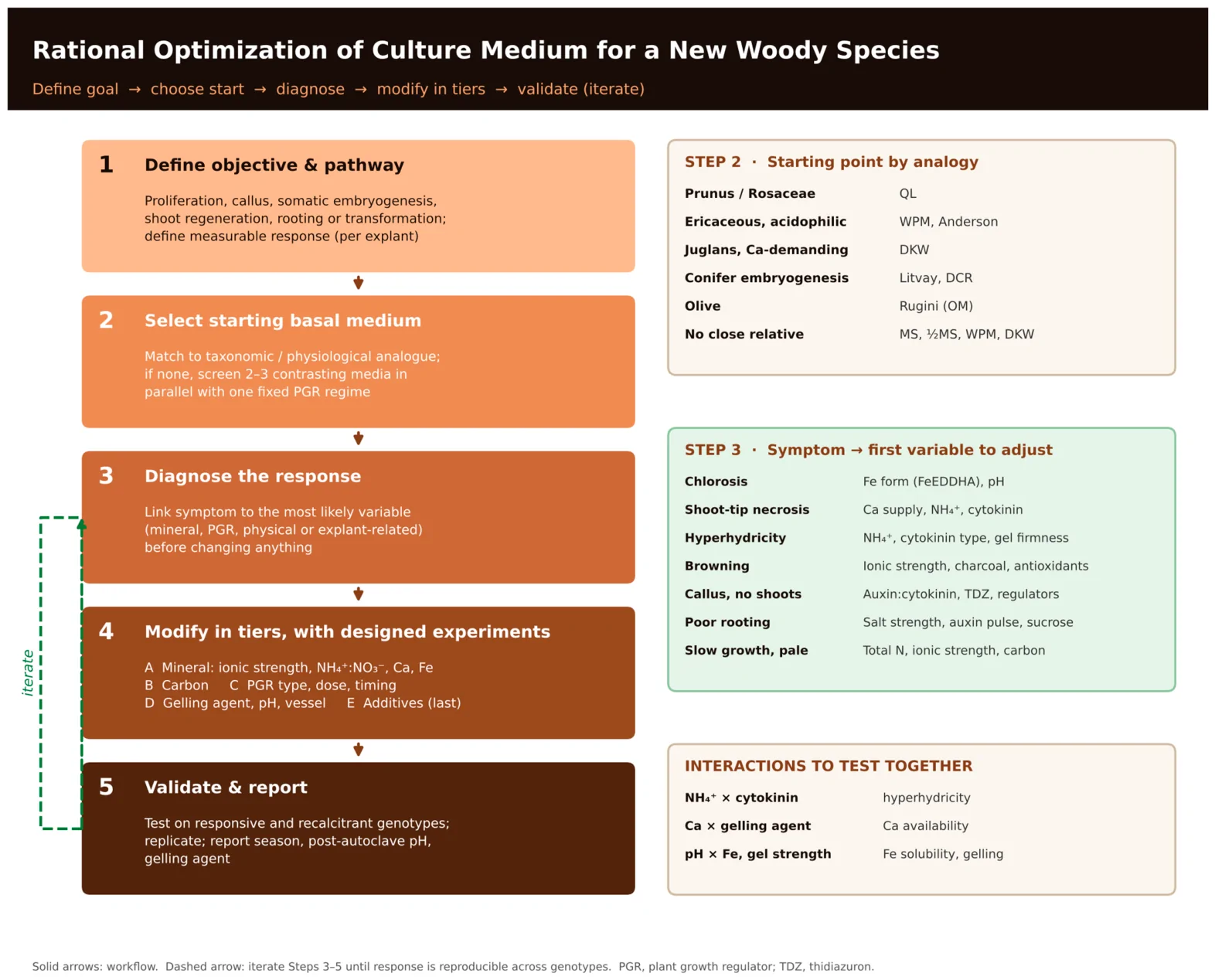
Decision scheme for the rational selection and modification of a culture medium for a new woody species or a recalcitrant genotype. The workflow (**left**) moves from definition of the developmental objective, through selection of a starting basal medium by taxonomic or physiological analogy and diagnosis of the observed response, to tiered modification using designed experiments and validation across genotypes; Steps 3–5 are repeated until the response is reproducible. The panels (**right**) give examples of starting media for different plant groups (Step 2), common symptoms and the variable to adjust first (Step 3), and variable interactions that should be tested together rather than one at a time. PGR, plant growth regulator; TDZ, thidiazuron.

Section 4 and Section 5 describe the formulations and components that define woody-species media. The practical question for a laboratory starting work with a new species, or with a genotype that fails on established protocols, is how to use this information. Optimization is most efficient when it is treated as a staged decision process rather than a search through all possible formulations (Figure 5). The process starts from a defined objective and an informed choice of starting medium, uses the observed symptoms to decide which variable to change, modifies variables in a deliberate order using designed experiments, and ends with validation across genotypes. The logic of each step is outlined below.

Step 1: define the objective and developmental pathway. Media that are optimal for one stage rarely suit the next. Establishment, shoot proliferation, callus induction, somatic embryo induction and maturation, regeneration after transformation, and rooting place different demands on mineral nutrition, carbon supply, and plant growth regulators (Section 5.5.4). This is why stage-specific medium series, such as those described in Section 4.7, are often more effective than a single formulation carried through all stages. The objective should be paired with a quantitative response variable, for example the proportion of explants forming shoots, shoots per explant, a shoot-quality score, or rooting percentage, recorded per explant rather than per vessel so that later comparisons are statistically interpretable.

Step 2: select a starting medium by analogy rather than by default. The most defensible starting point is the medium developed for the closest relative or ecophysiological analogue of the target: QL medium for *Prunus* and other Rosaceae, WPM for ericaceous shrubs and many other woody genera, Anderson’s medium for acidophilic species, DKW for *Juglans* and other calcium-demanding crops, and Litvay or DCR media for conifer somatic embryogenesis (Section 4). When no close relative has been studied, a small parallel screen is more informative than adopting MS by default. We suggest 2–3 media that differ in the variables that most often limit woody cultures, namely total ionic strength, ammonium-to-nitrate ratio, and calcium supply: for example, MS, half-strength MS or WPM (lower ionic strength), and DKW or QL (higher calcium or reduced total nitrogen) [5,22,23]. All treatments should share the same PGR regime, sucrose concentration, gelling agent, and pH, so that differences can be attributed to the mineral formulation. Because published formulations differ in salt hydrates and iron sources, compositions should be compared on a molar basis, as noted for Table 1.

Step 3: diagnose the response before changing variables. Observed symptoms point to the most probable cause (Figure 5, Step 3). Interveinal chlorosis points first to iron availability and pH, and suggests checking post-autoclave pH and substituting FeEDDHA for FeEDTA [32]. Shoot-tip and leaf-margin necrosis point to calcium supply, which can be addressed by raising calcium, reducing ammonium or cytokinin, and checking whether the gelling agent is limiting calcium availability [27,31]. Glassy, translucent shoots (hyperhydricity) point to a combination of high ammonium, high or highly active cytokinin, soft gels, and humid vessel atmospheres, and may respond to reduced ammonium, replacement of BAP or TDZ with meta-topolin, firmer gels, or better ventilation [19,20]. Browning points to phenolic oxidation and is managed through reduced ionic strength, activated charcoal, antioxidants, and frequent transfer (Section 3.2). Callus that fails to regenerate points to the auxin and cytokinin regime or to a lack of competence, and calls for a cytokinin-dominant medium, a more active cytokinin, reduced auxin carry-over, or morphogenic regulators (Section 5.5.4 and Section 7.5). Poor rooting often reflects excessive salt or nitrogen or an unsuitable auxin regime and is commonly addressed by lowering salt strength and applying an auxin pulse. Diagnosis is probabilistic because symptoms overlap, so only one or two changes should be tested at a time.

Step 4: modify variables in tiers using designed experiments. Variables are best adjusted in an order that runs from those that set the physiological baseline to those that fine-tune it. Tier A is mineral composition (total ionic strength, NH_4_^+^:NO_3_^−^ ratio, calcium, and iron form), because it conditions the response to everything else [7]. Tier B is carbon source and concentration. Tier C is the type, concentration, and timing of PGRs, ideally explored as a grid of one auxin and one cytokinin at three to four levels each. Tier D covers physical factors: gelling agent, pH and buffering, and vessel gas exchange. Tier E covers undefined organic additives, which are tested last because their composition is poorly defined and they can mask the effects of defined components. Within a tier, factorial or response-surface designs are preferable to one-factor-at-a-time testing because they reveal interactions that sequential tests miss, such as ammonium × cytokinin in hyperhydricity, calcium × gelling agent in calcium availability, pH × iron form, and activated charcoal × PGR removal (Section 5.6). Work on hazelnut, *Prunus* rootstocks, and *Actinidia* illustrates how such designs can be applied to macronutrient composition [123,160,162,185] (Section 7.1). The best level of each tier should be carried forward before the next is opened, and an earlier tier should be revisited if later changes alter the response.

Recalcitrant genotypes. When a genotype fails on a medium that works for others, medium modification should be combined with changes to the explant rather than pursued in isolation. Priority should go to the physiological state of the explant: juvenile or rejuvenated tissue, collection in a responsive season, and control of phenolics and contamination (Section 3.2, Section 3.3, Section 3.4 and Section 3.5). Optimization should then be carried out on a panel that includes at least one responsive and one recalcitrant genotype, so that the resulting formulation is not tuned to a single genotype and genotype × medium interactions are visible [12]. For recalcitrant material, the options with the greatest effect are usually a more active or alternative cytokinin (TDZ, meta-topolin), staged transfer between media of different PGR content, and morphogenic regulators (Section 7.5). If the response remains poor after Tiers A–E have been explored, the barrier is likely developmental rather than nutritional, and further medium refinement will show diminishing returns [11,12,13].

Step 5: validate and report. A formulation should be confirmed in independent experiments with adequate replication, on additional genotypes, and over more than one subculture, because early responses are not always sustained. Reporting season, explant source, post-autoclave pH, gelling agent brand, and the complete PGR regime allow other laboratories to reproduce the protocol and builds the datasets needed for the data-driven approaches described in Section 7.1.

Hypothetical example. Consider a newly collected woody species with no published protocol, whose shoots on MS medium show leaf-margin necrosis and glassy tissue. A parallel screen of MS, half-strength MS, WPM, and DKW indicates that necrosis is reduced on the calcium-rich DKW, but hyperhydricity persists. A Tier A factorial varying ammonium and calcium is then followed by a Tier C comparison of BAP and meta-topolin at three concentrations, and finally by a comparison of agar and gellan gum. The resulting medium is tested on three additional genotypes before it is adopted as the working protocol.

## 6. Applications and Impacts of Optimized Media in Woody Plant Tissue Culture

The basal media described in Section 4, the component-level considerations discussed in Section 5, and the recalcitrance-management strategies outlined in Section 3 ultimately serve practical purposes that extend beyond protocol development. Optimized media support three major application areas in woody plant biotechnology: conservation of rare and endangered woody species, commercial micropropagation and horticultural production, and genetic transformation and genome editing. This section briefly examines how woody-targeted media and their associated modifications enable these applications.

### 6.1. Conservation of Rare and Endangered Woody Species

Plant tissue culture provides a valuable set of tools for conserving woody species threatened by habitat loss, disease, climate change, and overexploitation. In vitro propagation supports the multiplication of limited source material, the establishment of disease-free clonal lines, and long-term germplasm preservation through slow-growth storage, cryopreservation, or synthetic seed approaches [186]. For woody species, where seed-based regeneration may be slow, irregular, genetically variable, or unavailable from rare genotypes, in vitro approaches can preserve valuable individuals that might otherwise be lost.

The choice of basal medium has direct consequences for conservation outcomes. For example, multiplication of *Quercus robur* trees up to 800 years old was achieved on agar-solidified WPM with BAP, but only about half of the oldest donor trees sustained cultures beyond six months and a temporary immersion bioreactor gave no multiplication advantage on WPM [133], illustrating that medium choice can make recovery of mature genotypes feasible while outcomes remain strongly genotype-dependent. Similarly, conservation-oriented regeneration systems in wild or underutilized woody fruit species, such as *Pyrus* spp., often rely on QL- or related woody-targeted formulations that better support shoot proliferation and regeneration than general-purpose media [114]. Conservation applications also benefit from the antioxidant supplementation, phenolic-management strategies, and seasonal-collection guidance discussed in Section 3, because material collected from natural populations is often physiologically variable and prone to browning or contamination.

However, in vitro conservation should not be viewed simply as clonal multiplication. For threatened species, conservation programs must also consider genetic diversity, source-population representation, pathogen indexing, and long-term stability of stored material. Optimized woody-species media provide the technical foundation for culture establishment and propagation, but conservation success depends on integrating tissue culture with broader genetic and ecological conservation strategies.

### 6.2. Commercial Propagation and Horticultural Industry Benefits

Although this review emphasizes media formulation from a scientific and technical perspective, many of the same protocols support commercial micropropagation of ornamental, fruit, nut, and forestry species. Commercial micropropagation benefits nursery and horticultural industries by enabling rapid multiplication of selected genotypes, production of uniform and true-to-type planting material, recovery of disease-free stock through meristem or shoot-tip culture, and propagation of species or cultivars that are difficult to multiply efficiently by conventional methods.

Translation from research-scale protocols to commercial production usually requires additional optimization. Media must be adapted for scale, cost, labor efficiency, and consistency. This often involves reducing unnecessary additives, replacing expensive reagents where possible, optimizing subculture intervals, selecting appropriate gelling agents or liquid-culture systems, and adapting protocols to temporary immersion or other semi-automated platforms. The basal media discussed in Section 4, particularly WPM, DKW, Anderson’s medium, and QL medium, provide starting frameworks, but commercial laboratories typically refine plant growth regulator concentrations, sucrose levels, pH, vessel type, and culture environment for each cultivar and production stage.

Woody crops commonly represented in commercial micropropagation include ornamental shrubs such as *Rhododendron*, *Rosa*, *Hydrangea* [187], and *Lagerstroemia*; berry crops such as *Vaccinium*, *Rubus*, and *Ribes*; fruit and nut tree rootstocks such as *Prunus*, *Pyrus*, *Malus*, *Juglans*, and *Corylus*; and forestry or plantation species such as *Eucalyptus*, *Populus*, and conifers. For these systems, the practical lesson is that basal-medium choice is not arbitrary. Selection of WPM, DKW, Anderson’s medium, QL medium, or a species-specific formulation should be guided by the physiological and taxonomic similarity of the target species to the species for which each medium was originally developed, followed by empirical optimization for genotype, explant source, culture stage, and production scale.

### 6.3. Genetic Transformation and Gene Editing of Woody Species

Genetic transformation and genome editing of woody species depend critically on efficient in vitro regeneration. Delivery of transgenes or editing reagents into woody cells has limited practical value unless transformed or edited cells can be regenerated into whole plants. Regeneration competence, and therefore medium optimization, is often one of the major rate-limiting steps in woody-species transformation pipelines [55,125].

Transformation in woody species is dominated by *Agrobacterium tumefaciens*-mediated delivery, with biolistic delivery used selectively for recalcitrant tissues and protoplast- or ribonucleoprotein-based delivery explored where DNA-free or transgene-free editing is desired [188,189]. The regeneration media used during transformation generally follow the species-specific patterns discussed in Section 4: MS-based or modified MS systems remain common in some *Citrus*, *Prunus*, and grape protocols; WPM is widely used for *Populus* and other forest hardwoods; Anderson’s medium or WPM is used for *Vaccinium* and *Rubus*; DKW is important for *Juglans*, *Corylus*, and other calcium-demanding woody systems; and QL-based formulations remain relevant for *Prunus*, *Pyrus*, and rose. The plant growth regulator regimes used during transformation typically follow the direct organogenesis, indirect organogenesis, or somatic embryogenesis pathways described in Section 5.5.4, with additional attention to antioxidants, antimicrobial agents, ethylene inhibitors, and gelling systems that reduce stress after wounding or *Agrobacterium* co-cultivation.

CRISPR/Cas-based genome editing has now been reported in a growing number of woody crops, including apple, citrus, grape, kiwifruit, pear, poplar, cacao, coffee, pomegranate, and others [188,189,190]. Major editing targets include disease-susceptibility genes such as *MLO* and *LOB1*, pigment or visible-marker genes such as *PDS* and *DFR*, and genes associated with fruit quality, growth habit, flowering, and stress tolerance. For each species, editing success depends not only on guide RNA design or delivery efficiency but also on the regeneration system that recovers edited cells as plants. Thus, the basal salt formulation, PGR regime, carbon source, iron form, gelling agent, pH, and anti-browning or antimicrobial amendments discussed in this review directly influence the feasibility of woody-species genome editing.

As editing technologies continue to evolve toward base editing, prime editing, transient delivery, and DNA-free editing, regeneration remains the central bottleneck for many woody species. Where conventional media optimization has reached practical limits, emerging strategies such as statistical and machine-learning-assisted media optimization and morphogenic-regulator-assisted regeneration are being explored, as discussed in Section 7. These approaches are likely to be most effective when built on the physiological principles summarized in Section 3, Section 4 and Section 5 rather than used as substitutes for rational medium design.

## 7. Innovations and Emerging Directions in Woody Plant Tissue Culture

The basal media described in Section 4 and the component-level principles discussed in Section 5 represent the established chemistry of woody-species tissue culture. Beyond these classical formulations, several emerging approaches are beginning to reshape how media are designed and how recalcitrant woody species are regenerated. This section highlights five active areas: statistical and machine-learning-assisted media optimization, nanoparticle-based amendments, temporary immersion systems, natural extracts and biostimulants, and morphogenic-regulator-assisted regeneration. These approaches are not equivalent and are grouped here by the type of limitation they address. Statistical and machine-learning-assisted optimization, nanoparticle amendments, and natural extracts (Section 7.1 and Section 7.2, and Section 7.4) change or search the chemical composition of the medium. Temporary immersion systems (Section 7.3) change how the medium and gas environment are delivered to the tissue, altering water availability, aeration, and nutrient access without changing the formulation. Morphogenic regulators (Section 7.5) act on the developmental competence of the explant rather than on the medium. Their evidence base and practical limits differ and are compared in Section 7.6.

### 7.1. Statistical and Machine-Learning-Assisted Media Optimization

The empirical optimization of media for recalcitrant woody species has historically relied on one-factor-at-a-time experimentation, which is slow, labor-intensive, and often fails to capture interactions among nutrients, plant growth regulators, explant type, and genotype. Statistical experimental design and machine-learning-assisted optimization provide more efficient alternatives by allowing multiple variables to be evaluated simultaneously and by identifying interaction effects that are difficult to detect through conventional empirical screening.

The clearest examples come from hazelnut (*Corylus avellana*) and *Prunus* rootstock systems. In DKW-based hazelnut cultures, response surface methodology and data-mining approaches were used to evaluate modified macronutrient combinations and identify nutrient factors associated with shoot quality and multiplication [123,162]. In *Prunus* G × N15 rootstock, artificial neural networks combined with genetic algorithms were used to refine QL macronutrient composition and improve proliferation-related parameters [160]. Similar factorial approaches have also been applied to *Actinidia arguta*, where MS macronutrients were grouped into independent factors for optimization [185].

The broader lesson is that woody-species media should increasingly be treated as multi-factor systems rather than fixed recipes. Data-driven optimization is particularly valuable when genotype dependence is strong, when the number of possible nutrients and PGR combinations is large, or when transformation pipelines require reliable regeneration from specific elite genotypes. However, these approaches still require high-quality experimental datasets, careful biological interpretation, and validation across genotypes and laboratories before optimized formulations can be generalized. Such designs are most useful when embedded in the staged workflow outlined in Section 5.8, in which the choice of starting medium and the diagnosis of observed symptoms determine which factors are worth including in the design.

### 7.2. Nanoparticle-Based Delivery and Elicitation

Engineered nanoparticles, including silver, zinc oxide, copper, titanium dioxide, and carbon-based nanomaterials, have been explored in plant tissue culture as antimicrobial agents, anti-browning agents, nutrient sources, and stress-modulating amendments. Their biological effects arise from high surface area, tunable release kinetics, and interactions with reactive oxygen species, microbial cells, and plant tissues.

In woody-species culture, silver nanoparticles have been reported to improve explant disinfection and root system development in gray poplar (*Populus tremula* × *P. alba*) [191]. Zinc oxide nanoparticles have been tested in olive and date palm cultures, with reported effects on shoot growth, callus proliferation, rooting, and micropropagation efficiency [192,193]. These examples suggest that nanoparticles may provide useful tools for managing contamination, oxidative stress, and nutrient availability in recalcitrant woody systems.

However, nanoparticle applications should be interpreted cautiously. Responses are highly dose-dependent, species-specific, and influenced by particle size, coating chemistry, medium composition, and exposure duration. Toxicity thresholds remain incompletely characterized for many woody species, and regulatory or environmental concerns may constrain commercial deployment. Therefore, nanoparticle-based approaches are best viewed as promising experimental tools rather than established standard components of woody-species culture media.

### 7.3. Temporary Immersion Bioreactor Systems

Temporary immersion bioreactor systems (TIS) periodically immerse explants in liquid medium and then drain the culture, combining improved nutrient access with better aeration than continuous liquid culture [179]. Common platforms include RITA^®^, Plantform™, and custom ebb-and-flow systems. Although temporary immersion systems are not media formulations in the narrow chemical sense, they strongly influence how media components are delivered and therefore interact with basal salt composition, sucrose level, plant growth regulators, and anti-hyperhydricity strategies.

For woody species, temporary immersion systems are particularly useful in three contexts. First, conifer somatic embryogenesis systems based on Litvay or DCR media can benefit from liquid or temporary immersion culture during proliferation, maturation, or germination stages, provided that immersion frequency and aeration are carefully controlled [194]. Second, the benefit of temporary immersion is not universal: in *Quercus robur* trees up to several centuries old, a RITA^®^ bioreactor did not increase multiplication relative to agar-solidified WPM, although with modified QL medium it outperformed vertically oriented agar cultures [133]. Third, temporary immersion systems have been applied to large-scale propagation of woody crops and ornamentals, including blueberry, coffee, and selected forest or ornamental species.

Beyond conifer and *Quercus* examples, temporary immersion has been validated across several other woody genera. In *Eucalyptus*, RITA^®^-type and related automated bioreactors have produced normal shoot cultures with reduced callus formation relative to semi-solid media, and eucalyptus alone accounts for roughly a third of the published tree-species TIS literature, making it one of the most extensively optimized woody taxa for this technology [195,196]. In *Prunus*, SETIS™ and GreenTray^®^ bioreactor systems have been applied to almond, peach, and sweet cherry rootstocks (e.g., GF677, Gisela 6), consistently showing faster shoot elongation, higher biomass accumulation, and improved rooting compared to semi-solid culture, alongside reduced hyperhydricity relative to solid medium in comparative *Prunus*/*Malus* trials [197,198,199,200]. In *Populus*, a two-vessel temporary immersion bioreactor (TIB) system enabled the first reported high-multiplication-rate micropropagation protocol for dendroenergetic poplar hybrids under photomixotrophic conditions, and TIS has more recently been extended to specialized phenolic-compound bioproduction in *Populus nigra* shoot cultures. In *Coffea* and *Hevea*, temporary immersion has been central to somatic embryogenesis scale-up: optimizing immersion frequency and duration improved regeneration, morphology, and embryo water/mineral status in *Coffea arabica*, supported Robusta embryo densities on the order of 200,000–400,000 per liter within two months, and—in one of the earliest reported woody-plant applications of the technique—improved somatic embryogenesis efficiency in *Hevea brasiliensis* [197,198,199,200,201,202,203,204,205,206].

The major advantages of temporary immersion systems include improved nutrient uptake, reduced labor, scalability, and potential improvement in shoot or embryo quality. Their main limitations are equipment cost, greater technical complexity, and the need to re-optimize protocols when transitioning from semi-solid to liquid-based culture. In many woody species, immersion duration, immersion frequency, vessel ventilation, explant density, and medium composition must be optimized together to avoid hyperhydricity or tissue stress.

### 7.4. Natural Extracts and Biostimulants

Plant-derived natural extracts and biostimulants, including coconut water, banana pulp, malt extract, yeast extract, algal extracts, and botanical infusions, continue to be used in plant tissue culture as sources of organic nutrients, natural hormones, amino acids, minerals, and growth-promoting compounds. Interest in these materials is driven by cost, local availability, sustainability, and the observation that complex biological extracts can sometimes improve growth or regeneration beyond what is achieved with defined components alone.

In woody-species culture, coconut water remains the best-known natural additive and functions mainly as a complex source of natural cytokinin, sugars, amino acids, and minerals, as discussed in Section 5.6 [178]. Banana pulp, malt extract, yeast extract, and seaweed-derived extracts have also been used in selected woody or perennial systems, particularly in somatic embryogenesis, rooting, or low-cost micropropagation contexts [207]. These additives may be especially attractive in commercial or regional laboratories where reducing medium cost is important.

The principal limitation of natural extracts is reproducibility. Their composition varies with species, cultivar, maturity stage, harvest conditions, processing method, and storage. For this reason, natural extracts are less suitable for mechanistic studies or protocols requiring precise cross-laboratory reproducibility unless their composition is characterized and compared directly with defined alternatives. In research-grade woody-species protocols, defined supplements such as specific amino acids, purified cytokinins, or known osmotic agents are generally preferable. Natural extracts remain useful when their effects are empirically validated, economically justified, and appropriately standardized.

### 7.5. Morphogenic-Regulator-Assisted Regeneration

Morphogenic-regulator-assisted regeneration represents one of the most important recent developments for overcoming genotype-dependent recalcitrance. Morphogenic regulators are developmental genes or transcription factors that promote embryogenic, meristematic, or regenerative competence. Rather than relying solely on exogenous PGRs and medium composition, this strategy supplies developmental signals genetically or transiently to increase the capacity of explants to regenerate.

Foundational work in monocots showed that expression of BABY BOOM and WUSCHEL2 expanded the range of transformable maize genotypes and explant types [91]. Subsequent work demonstrated that a GRF4-GIF1 fusion protein improved regeneration in several plant systems [65]. These approaches have encouraged similar efforts in woody species. In poplar, selected developmental regulators, including WOX- and GRF-related genes, have been reported to enhance de novo shoot regeneration [208]. In apple, *MdBBM1* expression improved transformation efficiency in ‘Royal Gala’, and WOX-related approaches have been explored for regeneration from recalcitrant tissues [112]. In citrus, morphogenic-regulator-assisted and hairy-root-coupled strategies have been investigated to address genotype dependence and improve transformation efficiency [209,210]. PLT5 and combinations of developmental regulators have also been reported to improve regeneration or transformation outcomes across diverse plant systems [211].

The implications for media formulation are significant but still evolving. Morphogenic regulators may reduce dependence on complex cytokinin- or auxin-heavy media, or they may potentiate the effects of classical PGRs. However, regeneration outcomes still depend strongly on basal medium chemistry, explant physiology, selection conditions, and culture environment. In addition, constitutive expression of morphogenic regulators can cause abnormal development, pleiotropic effects, or difficulties in recovering normal plants, so inducible, transient, tissue-specific, or excisable systems may be needed for practical deployment.

Thus, morphogenic-regulator-assisted regeneration should be viewed as complementary to, rather than a replacement for, rational media optimization. The most effective future systems will likely combine optimized basal media, pathway-specific PGR regimes, stress-mitigation amendments, and controlled expression or delivery of morphogenic regulators. In this sense, the boundary between chemical supplementation and genetic supplementation is becoming less rigid, and progress in woody-species regeneration will increasingly depend on integrating both approaches [189,211].

### 7.6. Comparison, Practical Limitations, and Reproducibility

The approaches above address different limitations and should not be treated as interchangeable (Table 4). Only the first three alter the chemical composition of the medium; temporary immersion changes the delivery of medium and gas, and morphogenic regulators change the explant. Combining them is plausible, but their effects have rarely been tested together in woody species.

The evidence supporting these approaches is uneven. Statistical optimization offers the most transferable method, but the woody-species examples come from a few systems, typically one or a few genotypes, and optimized formulations have seldom been tested across genotypes or laboratories. Reports on nanoparticles and natural extracts are mostly single-species studies in which the effect depends on dose, particle properties, or extract batch, so reproducibility cannot be assumed. Temporary immersion has the broadest validation, but results obtained with one vessel design, immersion frequency, or medium do not transfer automatically to another species or system. Morphogenic regulators are the most promising route to genotype-independent regeneration, yet in woody species they remain at an early stage, depending on transformation, and can cause abnormal development when expressed constitutively. A result obtained with one genotype in one laboratory should therefore be treated as a hypothesis for the next genotype rather than as a general protocol. Studies would be more useful if they included more than one genotype, reported independent replicate experiments, and published negative or genotype-specific results.

## 8. Summary

Plant tissue culture media for woody species occupy a distinctive niche in plant biotechnology. Whereas many herbaceous systems use MS medium as a common starting formulation, woody plant tissue culture has developed a diversified portfolio of basal media, including QL medium for *Prunus* and related Rosaceae, WPM for ericaceous shrubs and many other woody genera, Anderson’s medium for acidophilic species, DKW medium for walnut and other calcium-demanding woody crops, and species-specific formulations such as Rugini Olive Medium for olive, Litvay and DCR media for conifer somatic embryogenesis, C2D medium for grapevine, and Hasegawa’s stage-specific media for rose [5,24,25,26,27,28,29,30,119,148,174]. This diversification reflects the biological complexity of woody species in vitro, including phenolic oxidation, endogenous contamination, mature-tissue recalcitrance, juvenility and phase change, seasonal variation, hyperhydricity, and genotype-dependent variability.

This review emphasizes three major points. First, woody-targeted media are best understood as compromise solutions to specific physiological and developmental constraints rather than as interchangeable salt mixtures. Basal-medium selection should therefore be guided by the taxonomic, physiological, and developmental similarity of the target species to the systems for which each medium was originally developed. Second, plant growth regulator strategies in woody-species protocols require a pathway-dependent framework. Callus induction, indirect organogenesis, direct organogenesis, somatic embryogenesis, and rooting each require distinct combinations of auxin type, cytokinin type, concentration, and exposure duration; the classical auxin-to-cytokinin ratio remains useful but does not fully explain the diversity of responses observed in woody systems [6]. Third, although many woody-targeted media developed in the 1970s and 1980s remain widely used, recent approaches, including statistical and machine-learning-assisted optimization, nanoparticle-based amendments, temporary immersion systems, natural extracts, and morphogenic-regulator-assisted regeneration, are expanding the tools available for recalcitrant species.

For researchers establishing new protocols in woody plants, several practical principles emerge. Basal-medium selection should begin with the known physiology of the target species and the original objective of candidate media. PGR regimes should be matched to the intended developmental pathway and downstream application, especially when regeneration is required for transformation or genome editing. Common modifications, such as FeEDDHA substitution for chlorosis-prone cultures, meta-topolin for systems prone to cytokinin-associated disorders, gellan gum for improved gel consistency or calcium-sensitive systems, and MES buffering where pH drift is problematic, should be evaluated empirically rather than adopted automatically. In addition, recalcitrance barriers should be considered together, because sterilization, browning, maturity, hyperhydricity, and genotype dependence frequently interact. In practice, this suggests a staged approach (Figure 5, Section 5.9): defining the developmental objective, selecting a starting medium by physiological analogy or a small parallel screen, diagnosing symptoms before changing variables, modifying mineral, carbon, PGR, physical, and additive factors in that order using factorial designs, and validating the result across responsive and recalcitrant genotypes.

The conclusions of this review differ in how well they are supported. Reasonably well supported are the following: total nitrogen load, nitrogen form, ionic strength, and calcium supply are the main compositional variables that distinguish woody-targeted media from MS (Table 1 and Supplementary Table S1); explant physiological age and collection season strongly affect the response; and the requirements for auxin, cytokinin, exposure time, and phase sequence differ among shoot proliferation, adventitious organogenesis, somatic embryogenesis, and rooting (Table 3). Less well supported are the physiological mechanisms by which particular media benefit particular genera, which are inferred largely from empirical comparisons in single species; the independent contributions of ammonium, ionic strength, and calcium to hyperhydricity and shoot-tip necrosis, which have rarely been separated experimentally and sometimes give contradictory results [100]; the transferability of optimized formulations between genotypes and laboratories; and the practical value of nanoparticle, natural-extract, and morphogenic-regulator approaches in woody species (Table 4). These are the aspects of medium optimization that need further investigation most, preferably through multi-genotype, multi-laboratory experiments that report explant source, season, and culture conditions in full.

Several important challenges remain. Mature-tissue recalcitrance continues to limit regeneration and transformation of elite woody genotypes and is unlikely to be solved by medium optimization alone. The mechanistic basis for many empirical medium responses, including why specific nutrient profiles favor particular woody genera, remains incompletely understood. Cross-laboratory reproducibility is also limited by under-reporting of explant season, physiological status, post-autoclave pH, gelling-agent properties, and other variables that strongly influence culture outcomes. As genome editing moves toward more precise, transient, and DNA-free approaches, efficient regeneration will remain a central bottleneck. Future progress will depend on integrating rational medium design, improved reporting standards, data-driven optimization, and developmental-regulator-assisted regeneration. The woody-targeted media reviewed here will continue to provide the chemical foundation for that progress.

## Figures and Tables

**Table 1:** Compositional Comparison of Major Basal Media Used for Woody Plant Tissue Culture.

Component	MS (1962)	B5 (1968)	WPM (1980)	DKW (1984)	Anderson’s (1984)	QL (1977)	Rugini OM (1984)	Litvay (1981)	DCR (1985)
Macronutrients									
NH_4_NO_3_	1650	—	400	1416	400	400	412	1650	400
KNO_3_	1900	2500	—	—	480	1800	1100	1900	340
Ca(NO_3_)_2_·4H_2_O	—	—	556	1968	—	833.77	600	—	556
CaCl_2_·2H_2_O	440	150	96	149	440	—	440	22 *	85 *
MgSO_4_·7H_2_O	370	250	370	740	370	360	1500	1850 *	370
KH_2_PO_4_	170	—	170	265	—	270	340	340	170
K_2_SO_4_	—	—	990	1559	—	—	—	—	—
(NH_4_)_2_SO_4_	—	134	—	—	—	—	—	—	—
NaH_2_PO_4_·H_2_O	—	150	—	—	380	—	—	—	—
KCl	—	—	—	—	—	—	500	—	—
Micronutrients									
H_3_BO_3_	6.2	3.0	6.2	4.8	6.2	6.2	12.4	31.0 *	6.2
MnSO_4_·H_2_O	16.9 †	10.0	22.3 ‡	33.5	16.9	0.76	16.9 *	21.0 *	22.3
ZnSO_4_·7H_2_O	8.6	2.0	8.6	—	8.6	8.6	14.3	43.0*	8.6
Zn(NO_3_)_2_·6H_2_O	—	—	—	17.0	—	—	—	—	—
KI	0.83	0.75	—	—	0.30	0.08	0.83	4.15 *	0.83
Na_2_MoO_4_·2H_2_O	0.25	0.25	0.25	0.39	0.25	0.25	0.25	1.25 *	0.25
CuSO_4_·5H_2_O	0.025	0.025	0.25	0.25	0.025	0.025	0.25	0.50 *	0.025
CoCl_2_·6H_2_O	0.025	0.025	—	—	0.025 *	—	0.025	0.125 *	—
NiSO_4_·6H_2_O	—	—	—	0.005	—	—	—	—	—
Iron source									
FeSO_4_·7H_2_O	27.8	27.8	27.8	var.	55.6	27.8	27.8	27.8	var.
Na_2_EDTA·2H_2_O	37.3	37.3	37.3	var.	74.5	37.3	37.3	37.3	var.
FeNaEDTA	var.	var.	var.	36.7/var.	var.	var.	var.	var.	36.7/var.
Vitamins									
myo-inositol	100	100	100	100	100	100	100	100	200
Thiamine·HCl	0.1	10.0	1.0	2.0	0.4	0.4	0.5	0.1	1.0
Nicotinic acid	0.5	1.0	0.5	1.0	—	1.0	5.0	0.5	0.5
Pyridoxine·HCl	0.5	1.0	0.5	—	—	0.5	0.5	0.1	0.5
Glycine	2.0	—	2.0	2.0	—	—	2.0	2.0	2.0
Biotin	—	—	—	—	—	0.01	0.05	—	—
Folic acid	—	—	—	—	—	—	0.5	—	—
Other organics									
Adenine sulfate	—	—	—	—	80	—	—	—	—
Glutamine	—	—	—	—	—	—	2.19 *	—	—
Carbohydrate									
Sucrose (g/L)	30	20–30	20	30	30	30	—	30	30
Mannitol (g/L)	—	—	—	—	—	—	30–36	—	—
pH	5.7–5.8	5.5	5.6–5.8	5.7–5.8	4.5 *	5.6–5.8	5.7 *	5.7	5.7

Some media components are reported differently among sources because formulations may use anhydrous salts, hydrated salts, or equivalent chelated forms. Where possible, mineral components should be compared on an elemental or molar basis. Iron may be supplied either as FeSO_4_·7H_2_O plus Na_2_EDTA·2H_2_O or as FeNaEDTA, depending on the source formulation. Iron values should therefore be compared on an Fe molar basis when possible. † MS originally specified MnSO_4_·4H_2_O at 22.3 mg/L, equivalent to approximately 16.9 mg/L MnSO_4_·H_2_O. Modern catalog formulations commonly list the monohydrate form. ‡ WPM formulations may vary in manganese source and concentration; some commercial preparations list MnSO_4_·H_2_O at 22.3 mg/L. Anderson’s medium uses NaH_2_PO_4_·H_2_O rather than KH_2_PO_4_ and includes adenine sulfate. Commercial suppliers may offer modified variants. DKW originally provides zinc as Zn(NO_3_)_2_·6H_2_O, although some commercial preparations substitute ZnSO_4_·7H_2_O. QL calcium nitrate values vary among sources depending on whether calcium nitrate is reported as an anhydrous or hydrated salt and whether the original or modified QL recipe is used. Asterisks (*) indicate values that vary among published or commercial formulations. Original formulations and later modified or commercial versions are not always identical. Commercial preparations commonly list anhydrous salts (e.g., Ca(NO_3_)_2_, CaCl_2_, MgSO_4_, NaH_2_PO_4_), supply iron as FeNaEDTA, and may omit or add minor components (for example, CoCl_2_ in QL or NiSO_4_ in DKW). Values marked * and the footnoted salt forms should therefore be checked against the primary publication before the table is used to compare formulations. QL: 833.77 mg/L Ca(NO_3_)_2_·4H_2_O, the form listed in this table, is equivalent to 578.92 mg/L anhydrous Ca(NO_3_)_2_, the form given in some commercial QL preparations; both correspond to 3.53 mmol/L Ca^2+^. If 578.92 mg/L were read as the tetrahydrate, Ca^2+^ would be only 2.45 mmol/L, which would change the calcium and nitrogen values in Supplementary Table S1 and how QL compares with MS.

**Table 2:** Representative Woody Species, Explant Types, Basal Media, and Culture Responses Reported in Woody Plant Tissue Culture.

Species	Family	Explant	Basal Medium	PGRs	Stage/Response	Reference(s)
Temperate Fruit Trees						
*Malus × domestica* (apple), 11 *M. sieboldii* hybrid genotypes	Rosaceae	Nodal segments	MS, QL, WPM, or DKW compared (MS best)	BAP 4.44 µM + IBA 0.25 µM + GA_3_ 0.28 µM	SP; AP-resistant rootstocks	[110]
*Malus × domestica* (apple), three rootstocks	Rosaceae	Nodal segments	MS or WPM	BAP 0.5–1.5 mg/L; IBA 0.5–1.5 mg/L (RT)	SP; RT	[111]
*Malus × domestica* ‘Royal Gala’	Rosaceae	Leaf	MS	BA 5 mg/L + TDZ 1 mg/L + NAA 1 mg/L; MdBBM1 pre-expression	SR enhanced by MdBBM1	[112]
*Pyrus communis* ‘Bartlett’ and ‘Beurre Bosc’	Rosaceae	Nodal, leaf	DKW > QL > MS, WPM	BA-based, var.	SP on DKW; SR enhanced after DKW preconditioning	[113]
*Pyrus communis* var. *pyraster* (wild pear)	Rosaceae	Leaf	QL	BA 8.8 µM (TDZ caused hyperhydricity)	SR 40–64%; 30-d dark induction	[114]
*Prunus avium*, *P. persica, P. armeniaca* (cherry, peach, apricot)	Rosaceae	Meristem, nodal	QL	BAP + IBA, var.	Origin of QL medium for *Prunus*	[24]
*Prunus armeniaca* (apricot)	Rosaceae	Nodal, meristem tips	DKW, WPM, or modified QL	BA + IBA, var.	SP; cultivar-specific media requirements	[115,116]
*Prunus* G × N15 (peach × almond) rootstock	Rosaceae	Microshoots	YAS (ANN-GA-optimized)	Macronutrient ratios optimized; PGRs std.	RT	[117]
Eight *Prunus* genotypes (peach, plum, interspecific hybrids)	Rosaceae	Microshoots	MS, ½ MS, DKW, or NPM	IBA 15 µM continuous, 4-d pulse, or 1 mM 30-s dip	RT; DKW maximized size in 6 of 8 genotypes	[118]
*Vitis vinifera* (grape)	Vitaceae	Nodal segments	C2D	BA 1–5 µM	SP; origin of C2D medium	[119]
*Vitis vinifera*	Vitaceae	Nodal segments	MS, WPM, C2D, QL	var.	SP; medium effect by cultivar	[120]
Nut Trees						
*Juglans hindsii × J. regia* (Paradox walnut)	Juglandaceae	Nodal segments	DKW	BA 4.5 µM + IBA 5 nM	SP; origin of DKW medium	[27]
*Juglans regia* (English walnut)	Juglandaceae	Somatic embryos	DKW	2,4-D + BA for SE; kanamycin selection	*Agrobacterium*-mediated transformation of woody tree	[121]
*Juglans sigillata* (iron walnut)	Juglandaceae	Cotyledon	DKW	IBA 0.2 mg/L + TDZ 6.0 mg/L; 10-d dark	SR 71.11%; 6.88 shoots/explant	[122]
*Corylus avellana* (hazelnut)	Betulaceae	Nodal segments	DKW	BAP 4.4 µM + IBA, var.	SP; CHAID/RSM-optimized ions	[123]
*Castanea sativa* (sweet chestnut)	Fagaceae	Nodal segments	Modified GD or MS with ½ nitrates	BAP 0.2 mg/L; IBA 25–50 mg/L 24-h pulse (RT)	SP from mature trees; EST	[124]
Citrus						
*Citrus sinensis* ‘Hamlin’ (sweet orange)	Rutaceae	Epicotyl	MS	BA 13.2 µM + 2,4-D 4.5 µM + NAA 0.5 µM (pre-infection)	SR; *Agrobacterium* transformation up to 25%	[103]
*Citrus latifolia* (Tahiti lime)	Rutaceae	Nodal segments	MS (SP); DKW (RT)	BA 1.0 + NAA 0.01 mg/L (SP); NAA 1.5 mg/L (RT)	SP on MS; best RT on DKW	[125]
*Citrus sinensis × Poncirus trifoliata* (Carrizo citrange)	Rutaceae	Epicotyl	MS	BAP 2.0 mg/L + NAA 0.05 mg/L; pre-infection BA + 2,4-D + NAA	SR; *Agrobacterium* transformation up to 47%	[103]
Berry Shrubs						
*Vaccinium corymbosum* ‘Bluejay’ (highbush blueberry)	Ericaceae	Two-node stem	Anderson’s	Zeatin 13.68 µM + NAA 0.27 µM; ½ Anderson’s + IBA 0.49 µM (RT)	SP; RT	[126]
*Vaccinium ashei* ‘Pink Lemonade’ (rabbiteye-derived)	Ericaceae	Two-node stem	Anderson’s	Zeatin 9.12 µM + NAA 0.05 µM	SP	[126]
*Vaccinium corymbosum* (highbush blueberry)	Ericaceae	Leaf	WPM	CPPU 2.0 mg/L + 2iP 0.2 mg/L	CI 97%; SR 71%	[127]
*Vaccinium meridionale* (Andean blueberry)	Ericaceae	Microshoots	DKW > WPM, MS	Zeatin/2iP/mT/TDZ at 2.5 µM equimolar; ex vitro RT with 500 ppm NAA	SP best on DKW with zeatin or 2iP	[128]
*Vaccinium arboreum* (sparkleberry)	Ericaceae	Nodal segments	LP > DKW, WPM	Zeatin 2.28 µM + IBA 0.05 µM	SP; pH tolerance evaluated 5–8.5	[129]
*Rubus fruticosus, R. idaeus* (blackberry, raspberry)	Rosaceae	Leaf	Modified MS or Anderson’s	TDZ 0.5–1.0 mg/L + NAA 0.1 mg/L	SR from leaves, cotyledon	[130]
*Ribes nigrum* (black currant)	Grossulariaceae	Dormant buds	MS	BA 2.0 + IBA 0.5 + GA_3_ 0.1 mg/L (establishment); BA + IBA, var.	SP	[131]
Subtropical/Tropical Fruit						
*Mangifera indica* (mango)	Anacardiaceae	Nucellar tissue	MS (modified)	2,4-D 1.0–2.0 mg/L + BAP	SE; severe browning	[132]
Forest Hardwoods						
*Quercus robur* (pedunculate oak), trees up to 800 yr	Fagaceae	Epicormic shoots	WPM (modified QL compared)	BAP 0.8 mg/L	SP from mature tissue; genotype-dependent	[133]
Forestry/Poplar						
*Populus tremula × P. alba* (gray poplar)	Salicaceae	Young tissues	WPM	KT 0.10 + 2,4-D 1.00 mg/L (CI); TDZ 0.02 mg/L (SR)	CI > 85.7%; SR	[134]
*Populus trichocarpa* (model)	Salicaceae	Nodal segments, leaf	MS or WPM	BAP + NAA, var.; with morphogenic regulators	SP, SR, transformation	[135]
*P. angustifolia*, *P. balsamifera*, and *P. deltoides*	Salicaceae	Stem and petiole explants	n.s.	n.s.	CI, SR	[136]
Conifers						
*Pinus taeda* (loblolly pine)	Pinaceae	Immature zygotic embryos	½ LM (Litvay)	2,4-D 10 µM + BA 5 µM	SE initiation	[29,137]
*Pinus nigra* (Austrian pine)	Pinaceae	Immature zygotic embryos	DCR or MLV	2,4-D 2.25–9 µM + BA 5 µM	SE initiation 5–10%	[138]
*Pinus sylvestris* (Scots pine)	Pinaceae	Bud explants	MS /DCR	BAP, 2IP, NAA, IBA	CI	[61]
*Picea abies* (Norway spruce)	Pinaceae	Immature zygotic embryos	AE	2,4-D, BAP	SE	[139]
*Pseudotsuga menziesii* (Douglas-fir)	Pinaceae	Mature shoots	DCR	BAP	SP from mature trees	[30]
*Abies alba × A. cephalonica* (fir hybrid)	Pinaceae	Mature and immature zygotic embryos	SH > DCR, ½ LM	BA 4.4 µM	SE initiation 4.4–38.1%	[140,141]
Tropical Economic Woody Species						
*Coffea arabica* (Arabica coffee)	Rubiaceae	Leaf	MS or half-strength MS	2,4-D 4.5 µM + Kin 4.6 µM	Indirect SE	[142]
*Theobroma cacao* (cacao)	Malvaceae	Floral tissues (staminodes, petals)	DKW (modified)	TDZ 0.005 mg/L + 2,4-D 2.0 mg/L	Primary SE	[143]
*Olea europaea* (olive)	Oleaceae	Nodal segments	Rugini OM	Zeatin 1.0 mg/L (filter-sterilized) or 3–4 mg/L (autoclaved)	SP	[28]
*Aquilaria malaccensis* (agarwood)	Thymelaeaceae	Leaf	MS or WPM	BAP + IBA, var.	CI	[144,145]
*Camptotheca acuminata* (happy tree)	Nyssaceae	Nodal segments	WPM > MS, B5	BA 4.44 µM	SR	[146]
Woody Ornamentals and Medicinals						
*Rosa hybrida* ‘Baronesse’	Rosaceae	Nodal segments	QL	BAP 3.0 mg/L + NAA 0.5 mg/L	30.3 plantlets/explant	[147]
*Rosa hybrida* ‘Moneyway’ (rose rootstock)	Rosaceae	Nodal segments	QL	BAP + NAA, var.	SP without chlorosis	[32]
*Rosa hybrida*	Rosaceae	Nodal segments, leaf	MS	Stage-specific PGR profiles	EST, RT	[148]
*Rhododendron griffithianum*	Ericaceae	Shoot tip	Anderson’s	2iP 5 µM + IBA 1.0 µM	SP > 75%	[149]
*Rhododendron laetum × R. aurigeranum* (Vireya)	Ericaceae	Shoot tip	Modified Anderson’s	TDZ + 2iP, var.	SR	[150]
*Lagerstroemia indica* (crape myrtle)	Lythraceae	Nodal segments	MS	6-BA 1.0 mg/L + NAA 0.1 mg/L	SP, RT	[151,152]
*Lagerstroemia speciosa* (Pride of India)	Lythraceae	Nodal segments	MS	mT 5.0 µM + NAA 0.5 µM	SP, 24.3 shoots/explant	[153]
*Gardenia jasminoides*	Rubiaceae	Nodal segments	Economou and Spanoudaki	BAP, 2iP, Kin	SP	[154]
*Acer platanoides*	Sapindaceae	Nodal and apical segments	MS/DKW, WPM	var.	SP	[155,156]
*Acer macrophyllum*	Sapindaceae	Shoot explants	½ MS, DKW, WPM	TDZ, IBA	SP, RT	[81]
*Magnolia* spp.	Magnoliaceae	Axillary buds	MS	BAP, Zt	SP	[157]

PGR concentrations follow the form used in the original publications (mg/L or µM). Stage abbreviations: SP = shoot proliferation; SR = shoot regeneration; CI = callus induction; SE = somatic embryogenesis; RT = rooting; EST = in vitro establishment; n.s., not specified. var., variable (concentrations or combinations differ among the cited studies). AP, apple proliferation. PGR abbreviations: BA/BAP, 6-benzyladenine (6-benzylaminopurine); IBA, indole-3-butyric acid; NAA, 1-naphthaleneacetic acid; GA_3_, gibberellic acid; TDZ, thidiazuron; 2,4-D, 2,4-dichlorophenoxyacetic acid; Kin/KT, kinetin; 2iP, 2-isopentenyladenine; mT, meta-topolin; Zt, zeatin; CPPU, N-(2-chloro-4-pyridyl)-N′-phenylurea. Medium abbreviations: NPM, New Prunus Medium [118]; LM, Litvay medium [29]; C2D, Chee and Pool medium [119]; GD, Gresshoff and Doy medium. Other abbreviations: RSM, response surface methodology; CHAID, chi-squared automatic interaction detection; ANN-GA, artificial neural network–genetic algorithm; YAS, Yadollahi, Arab and Shojaeiyan medium [117]; LP, Long and Preece medium [129]; MLV, modified Litvay medium (Litvay et al. 1981) with full or half concentration of macro- and microelements [138]; AE, Arnold and Eriksson medium [139].

**Table 3:** Pathway-dependent plant growth regulator requirements for the main regeneration routes in woody species.

Pathway	Auxin–Cytokinin Balance and Typical Compounds	Concentration Considerations	Exposure and Sequence	Common Problems
Axillary shoot proliferation	Cytokinin-dominant (BAP, zeatin, meta-topolin) with little or no auxin; low NAA or IBA may be added	Lowest cytokinin level that sustains multiplication; high levels shorten internodes and favor hyperhydricity and shoot-tip necrosis	Continuous during multiplication subcultures; transfer to lower-cytokinin or cytokinin-free medium for elongation before rooting	Hyperhydricity, short or fasciated shoots, declining vigor over subcultures [20]
Adventitious shoot organogenesis (direct or via callus)	Cytokinin-dominant (TDZ, BAP, zeatin) with low auxin; the indirect route begins with an auxin-rich induction phase	TDZ is active at much lower concentrations than BAP or zeatin; excess TDZ causes callus, hyperhydricity, and poor elongation	Short TDZ pulse or limited induction phase, then transfer to BAP, zeatin, or meta-topolin for shoot development and elongation	Callus without shoots, somaclonal variation after long callus phases, poor elongation [20,114]
Somatic embryogenesis	High, persistent auxin (2,4-D, picloram) at induction; auxin reduced or withdrawn for embryo development	Optimum depends on auxin type and genotype; 2,4-D, picloram, and NAA should be tested in parallel [140]	Induction, proliferation, then maturation on auxin-free medium with ABA and osmotic agents such as maltose, then germination; prolonged auxin exposure blocks embryo development	Loss of embryogenic capacity, abnormal embryos, poor maturation [140,175]
Adventitious rooting	Cytokinin reduced or removed; single auxin, most often IBA (or NAA)	Dose-dependent: too little gives few roots, too much gives basal callus and inhibits root elongation	Brief auxin pulse for induction, then auxin-free medium for root expression and elongation; reduced-salt medium commonly used [2,25]	Basal callus, poor root elongation, low rooting of difficult-to-root species [2,25]

**Table 4:** Emerging approaches in woody plant tissue culture, the level at which each acts, and their practical limitations.

Approach	Acts on	Main Limitation Addressed	Evidence Base and Practical Limits
Statistical and machine-learning-assisted optimization	Medium composition (search method)	Interactions among nutrients, PGRs, and genotype	Examples in hazelnut, *Prunus* rootstocks, and *Actinidia* [123,160,162,185]; mostly single systems and small datasets; optimized media rarely validated across genotypes or laboratories
Nanoparticle amendments	Medium composition (additive)	Contamination, oxidative stress, nutrient delivery	A few species (poplar, olive, date palm) [191,192,193]; strongly dose-, size-, and coating-dependent; toxicity thresholds and regulatory aspects unresolved
Natural extracts and biostimulants	Medium composition (undefined additive)	Cost; supply of organic nutrients and growth factors	Mostly empirical, species-specific reports [178,207]; batch-to-batch variability limits reproducibility
Temporary immersion systems	Delivery of medium and gas environment (culture system)	Hyperhydricity, aeration, labor, and scale-up	Broadest validation (*Eucalyptus*, *Prunus*, *Populus*, *Coffea*, *Hevea*, conifer embryogenesis) [194,195,196,197,198,199,200,201,202,203,204,205,206]; equipment cost; medium and immersion regime must be re-optimized for each species and vessel type
Morphogenic regulators	Developmental competence of the explant (genetic)	Genotype dependence and recalcitrance	Early-stage results in poplar, apple, and citrus [65,91,112,208,209,210,211]; requires transformation, may cause abnormal development; inducible or excisable systems needed

## Data Availability

No new data were created or analyzed in this study. Data sharing is not applicable to this article.

## Supplementary Materials

The following supporting information can be downloaded at: https://www.mdpi.com/article/10.3390/plants15213366/s1. Table S1. Molar concentrations of mineral ions in major basal media used for woody plant tissue culture.
