# Supplementary material for: Culture Media for Woody Plant Tissue Culture: From Classical Formulations to Rational Optimization
Source: Plants (Basel). 2026 Oct 30;15(21):3366. doi: 10.3390/plants15213366 (PMC13765570; doi:10.3390/plants15213366)
Supplement: Supplementary file 1 [file plants-15-03366-s001.zip › plants-4613933-supplementary.pdf]

Supplementary Table S1. Molar concentrations of mineral ions in major basal media used for woody plant tissue culture

Concentrations are derived from the salt compositions in Table 1 of the main text and expressed in mM for macronutrients and µM for micronutrients, allowing direct comparison of nutrient supply independent of the salt form used.

Ion / parameter	MS (1962)	B5 (1968)	WPM (1980)	DKW (1984)	Anderson's (1984)	QL (1977)	Rugini OM (1984)	Litvay (1981)	DCR (1985)
**Nitrogen (mM)**									
NO_3^-	39.41	24.73	9.71	34.47	9.74	29.86	21.11	39.41	13.07
NH_4^+	20.61	2.03	5.00	17.69	5.00	5.00	5.15	20.61	5.00
Total inorganic N	60.02	26.76	14.70	52.16	14.74	34.86	26.26	60.02	18.07
NO_3^- : NH_4^+ (molar ratio)	1.91	12.19	1.94	1.95	1.95	5.98	4.10	1.91	2.62
**Other macronutrient ions (mM)**									
K^+	20.05	24.73	12.61	19.84	4.75	19.79	20.09	21.32	4.62
Ca^{2+}	2.99	1.02	3.01	9.35	2.99	3.53	5.53	0.15	2.93
Mg^{2+}	1.50	1.01	1.50	3.00	1.50	1.46	6.09	7.51	1.50
$H_2PO_4^-$ (P)	1.25	1.09	1.25	1.95	2.75	1.98	2.50	2.50	1.25
SO_4^{2-} (S)	1.73	2.19	7.45	12.15	1.83	1.60	6.34	7.88	1.66
Cl^-	5.99	2.04	1.31	2.03	5.99	—	12.69	0.30	1.16
Na^+	0.20	1.29	0.20	0.10	3.16	0.20	0.20	0.21	0.10
**Micronutrient ions (µM)**									
Fe (as Fe–EDTA)	100.0	100.0	100.0	100.0	200.0	100.0	100.0	100.0	100.0
Mn^{2+}	100.0	59.2	131.9	198.2	100.0	4.50	100.0	124	131.9
B (as H_3BO_3)	100.3	48.5	100.3	77.6	100.3	100.3	200.5	501.4	100.3
Zn^{2+}	29.9	6.96	29.9	57.1	29.9	29.9	49.7	149.5	29.9
Cu^{2+}	0.100	0.100	1.00	1.00	0.100	0.100	1.00	2.00	0.100
MoO_4^{2-} (Mo)	1.03	1.03	1.03	1.61	1.03	1.03	1.03	5.17	1.03
Co^{2+}	0.105	0.105	—	—	0.105	—	0.105	0.525	—
Ni^{2+}	—	—	—	0.019	—	—	—	—	—
I^-	5.00	4.52	—	—	1.81	0.482	5.00	25.0	5.00
**Summary**									
Total cation charge (meq/L)	49.85	32.12	26.83	62.33	21.89	34.97	48.68	57.45	18.58
Total anion charge (meq/L)	50.10	32.24	27.15	62.74	22.15	35.04	48.97	59.03	18.80
Ionic strength (mM)	56.20	36.41	38.94	87.04	28.34	41.59	66.78	73.2	24.79

1. Values were calculated from the mineral-salt concentrations listed in Table 1 of the main text (mg/L ÷ molecular weight of the stated salt form) and assume complete dissociation. Complexation, ion pairing and precipitation in the prepared medium are not accounted for, so values represent nominal formulation concentrations rather than ion activities.

2. Molecular weights used (g/mol): NH_4NO_3 80.04; KNO_3 101.10; $Ca(NO_3)_2 \cdot 4H_2O$ 236.15; $CaCl_2 \cdot 2H_2O$ 147.01; $MgSO_4 \cdot 7H_2O$ 246.47; KH_2PO_4 136.09; K_2SO_4 174.26; $(NH_4)_2SO_4$ 132.14; $NaH_2PO_4 \cdot H_2O$ 137.99; KCl 74.55; H_3BO_3 61.83; $MnSO_4 \cdot H_2O$ 169.02; $ZnSO_4 \cdot 7H_2O$ 287.56; $Zn(NO_3)_2 \cdot 6H_2O$ 297.49; KI 166.00; $Na_2MoO_4 \cdot 2H_2O$ 241.95; $CuSO_4 \cdot 5H_2O$ 249.69; $CoCl_2 \cdot 6H_2O$ 237.93; $NiSO_4 \cdot 6H_2O$ 262.85; $FeSO_4 \cdot 7H_2O$ 278.01; $Na_2EDTA \cdot 2H_2O$ 372.24; FeNaEDTA 367.05.

3. Only inorganic salts are included. Organic components (vitamins, myo-inositol, adenine sulfate, glutamine, carbohydrates) are excluded; adenine sulfate in Anderson's medium would add approximately 0.2 mM SO_4^{2-}.

4. Iron was calculated from $FeSO_4 \cdot 7H_2O$ + $Na_2EDTA \cdot 2H_2O$ where these are listed, and from FeNaEDTA at 36.7 mg/L for DKW and DCR, whose iron source is listed as variable. Na^+ includes sodium contributed by EDTA salts and Na_2MoO_4; SO_4^{2-} includes sulfate from $FeSO_4$ and micronutrient sulfates.

5. $H_2PO_4^-$ is shown as the nominal phosphate species; at pH 5.5–5.8 phosphate is present predominantly, but not exclusively, as $H_2PO_4^-$. Total cation and anion charge are given as a check on the calculation; small imbalances reflect rounding of molecular weights and non-charged species (B, EDTA) excluded from the sums.

6. Ionic strength was calculated as I = ½ Σ cizi2 over the macronutrient ions listed. For media marked as variable in Table 1 of the main text (e.g., Litvay, Rugini OM, QL), values reflect the formulation shown in that table and will change with the variant used.

7. "—" indicates the ion is not supplied by any listed salt in that formulation.
